# Quantitative Ethnobotany of Medicinal Plants Used by Indigenous Communities in the Bandarban District of Bangladesh

**DOI:** 10.3389/fphar.2018.00040

**Published:** 2018-02-06

**Authors:** Mohammad O. Faruque, Shaikh B. Uddin, James W. Barlow, Sheng Hu, Shuang Dong, Qian Cai, Xiaohua Li, Xuebo Hu

**Affiliations:** ^1^Laboratory of Drug Discovery and Molecular Engineering, Department of Medicinal Plants, College of Plant Science and Technology, Huazhong Agricultural University, Wuhan, China; ^2^National-Regional Joint Engineering Research Center in Hubei for Medicinal Plant Breeding and Cultivation, Huazhong Agricultural University, Wuhan, China; ^3^Medicinal Plant Engineering Research Center of Hubei Province, Huazhong Agricultural University, Wuhan, China; ^4^Ethnobotany and Pharmacognosy Laboratory, Department of Botany, University of Chittagong, Chittagong, Bangladesh; ^5^Department of Pharmaceutical and Medicinal Chemistry, Royal College of Surgeons in Ireland, Dublin, Ireland; ^6^Hubei Cancer Hospital, Wuhan, China

**Keywords:** ethnomedicinal plants, indigenous communities, quantitative analysis, Bandarban, Bangladesh

## Abstract

This study documents information on significant ethnomedicinal plants, which was collected from the traditional healers of three indigenous communities of Bangladesh. The documented data were quantitatively analyzed for the first time in this area. The information was obtained through open-ended, semi-structured questionnaires. The benefits, importance and coverage of ethnomedicine were expressed through several quantitative indices including Informant Consensus Factor (ICF), Use Value (UV), Frequency of Citation (FC), Relative Frequency of Citation (RFC) and Relative Importance Index (RI). The agreement of homogeneity between the present and previous studies and among the indigenous communities was evaluated using the Jaccard Index (JI). A total of 159 ethnomedicinal plant species, which were distributed in 132 genera under 62 families, were documented from 174 informants. Of these, 128 plants were native and 31 were exotic. Of a majority of documented species, herbs and leaves were the most utilized plant parts for the preparation of ethnomedicines (45.28%) whereas pastes (63.03%) were the most popular formulations. Among the documented species, the dominant families were the Asteraceae (14 species) and the Lamiaceae (12 species). The highest ICF value was 0.77 for digestive system disorders. Based on UVs, the five most commonly used ethnomedicinal plant species in the study area were *Duabanga grandiflora* (0.43), *Zingiber officinale* (0.41), *Congea tomentosa* (0.40), *Matricaria chamomilla* (0.33) and *Engelhardtia spicata* (0.28). The highest RFC was recorded for *Rauvolfia serpentina* (0.25). The highest RI value was calculated for both *Scoparia dulcis* and *Leucas aspera* (0.83). Importantly, 16 species were reported with new therapeutic uses and to our knowledge, 7 species described herein have never been ethnobotanically and pharmacologically studied, viz: *Agastache urticifolia, Asarum cordifolium, C. tomentosa, E. spicata, Hypserpa nitida, Merremia vitifolia* and *Smilax odoratissima*. The present study showed that traditional treatment using medicinal plants is still widespread in the study area. Documentation of new ethnomedicinal species with their therapeutic uses shall promote further phytochemical and pharmacological investigations and possibly, lead to the development of new drugs.

## Introduction

Plant species have long played important roles for humanity. The formal study of these plants has proven to be a powerful tool in understanding how different indigenous communities relate to natural resources, notably for medical and pharmaceutical applications (de Albuquerque and Hanazaki, 2009). Indeed, ethnomedicinal study has been a fundamental source for the discovery of natural and synthetic drugs (Fabricant and Farnsworth, 2001). Ethnobotanical knowledge continues to provide a starting point for many successful drug screening projects in recent years (Heinrich and Bremner, 2006). According to data from the World Health Organization (WHO), about 80% of the world's population, especially the rural people of developing countries, still primarily rely on traditional medicines (Islam, 2006). On the other hand, the origins of over 50% of all pharmaceutical drugs could be traced back to ethnomedicine (Van Wyk et al., 1997).

Bangladesh is home to 35 indigenous communities, covering about 2% of the total population, who reside in various hilly and remote areas. These communities have diverse cultural backgrounds and practice their own traditional ethnomedicine for primary healthcare (Khan et al., 2015). It has been reported that more than 80% of the Bangladeshi use herbal medicines for their primary healthcare, of which ethnomedicinal plants constitute a major component (Yusuf et al., 2009). Adequate documentation of such knowledge, and especially of traditional ethnomedicinal practices, is important because ethnomedicinal healers have a long association with herbs and their medicinal properties (Kabir et al., 2014).

Notably, ethnomedicinal knowledge is usually passed verbally from one generation to the next through family members (Nadembega et al., 2011), and most of this knowledge has not been formally documented (Asase et al., 2008). However, in recent years, there has been a continuous decline in traditional medicinal practices, because of reduced interest in the younger generation toward traditional treatment systems, coupled with rural depopulation, mass deforestation, and migrations of traditional medicinal healers to other jobs. These factors have contributed to the rapid loss of this rich knowledge (Kadir et al., 2013). In contrast, ethnomedicinal research has gained interest among the scientific community (Heinrich, 2000). Bangladesh is a small country, covering an area of 147,570 sq km but rich in plant diversity, with 5,327 plant species (Pasha and Uddin, 2013). However, only a small portion of these have been subjected to either phytochemical or pharmacological investigation.

A total of 12 indigenous communities live within the studied area (Uddin, 2014) of which three i.e., Chak, Marma, and Tanchayanga were selected for the present study. To maximize documentation, initial contacts were established with indigenous students and local people (notably the Karbari, or headmen) to identify the traditional healers of the selected communities. The main objective of the current study was to comprehensively document the ethnomedicinal information from the traditional healers of these three communities, toward building up a comprehensive database of medicinal plants and their traditional uses, as we have been documenting the ethnomedicinal practices from other indigenous communities for a number of years (Faruque and Uddin, 2011, 2014; Uddin et al., 2013, 2014; Rahman et al., 2016). We aimed to perform quantitative analysis of the documented data using quantitative ethnobotanical indexes. A secondary objective was to identify new ethnomedicinal plant species within the study area, which may represent a potential source for the discovery of new drugs.

## Materials and methods

### Study area

The Bandarban is a hilly district situated in South-Eastern Bangladesh with an area of 4479.03 sq. km., between 21°11′ and 22°22′ North latitudes and 92°04′−92°41′ East longitudes. It is bounded by the Rangamati district in the north, Myanmar in the south, Chin Province (Myanmar) and Rangamati district in the east, Chittagong and Cox's Bazar districts in the west. The economy of Bandarban is predominantly agricultural (61.95%), mainly through Jhum cultivation. Of lesser importance is the commercial sector (9.92%), service industries (8.12%) non-agricultural labor (7%) and miscellaneous others of 1% each or less (Banglapedia, 2003). Out of the entire district area, forests and rivers occupy about 2730.48 sq. km. (60.96%) and 3.16 sq. km. (0.07%), respectively. The annual average temperature of this district varies from a maximum of 37°C to a minimum of 12.5°C. Annual average rainfall is 3031 mm.

### Field study and data collection

**Figure 1:**
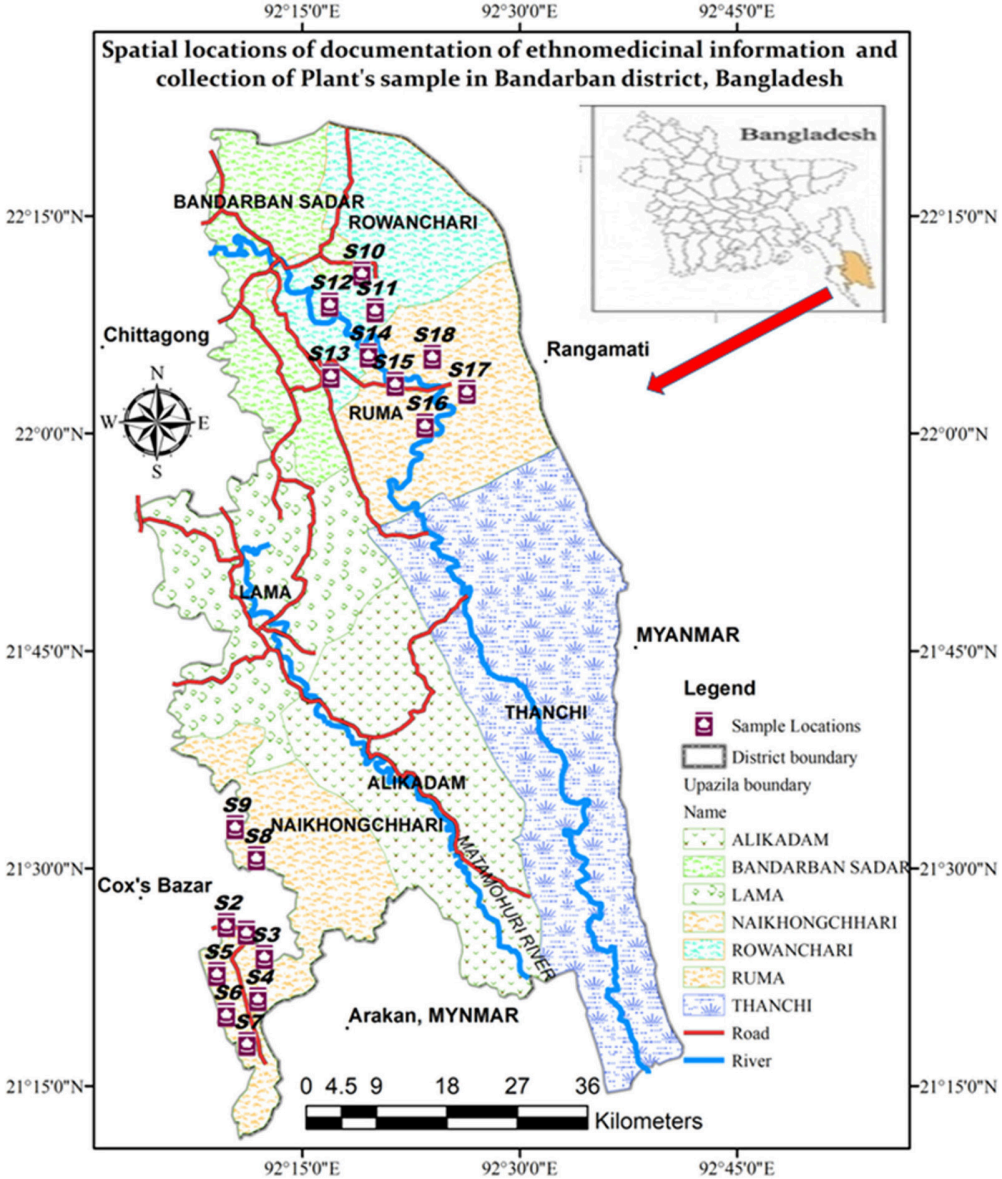
A map of the study area.

The field survey was carried out during both winter and summer seasons from January to April 2017. Three of the seven Bandarban district Upazilas were selected for the current study, namely Naikhyonchari, Rowangchari, and Ruma Upazilas (Figure 1). These three Upazilas were chosen due to their distance from cities, occupying some of the remotest areas of Bangladesh. A total of 12 indigenous communities live in the study area, including Bawm, Chak, Chakma, Khumi, Khyang, Lushai, Marma, Mro, Pangkhoa, Rakhaine, Tanchayanga, and Tripura (Uddin, 2014) Of these, three indigenous communities, namely, Chak, Marma, and Tripura, were included in the present study, as these communities were reported to use ethnomedicinal herbal practices heavily. Table 1 lists the details of visited areas along with their GPS readings. Ethnomedicinal data were documented through direct observation, field interview, group interview, and plant interview, by adopting open-ended and semi-structured question techniques (Martin, 1995; Alexiades and Sheldon, 1996). Audio and video recording was done throughout all interviews.

**Table 1:** Spatial locations of collected ethnomedicinal information/plants in Bandarban district, Bangladesh.

**Sample No**.	**Name of the area**	**Longitude (X)**	**Latitude (Y)**
S-1	Bichamara, Naikhonchhari Sadar	92°8′22.93149″	21°22′35.79366″
S-2	Bichamara, Naikhonchhari Sadar	92°8′57.4917″	21°21′21.50105″
S-3	Bichamara, Naikhonchhari Sadar	92°10′12.67734″	21°30′57.17177″
S-4	Chak Headman Para, Naikhonchhari, Bandarban	92°10′5.75284″	21°31′19.02916″
S-5	Sonaichari, Naikhonchhari, Bandarban	92°19′46.81″	21°9′27.23″
S-6	Kyang Para, Naikhonchhari, Bandarban	92°19′46.81″	21°9′27.23″
S-7	Kyang Para, Naikhonchhari, Bandarban	92°4′38″	21°24′52.15″
S-8	Baisari, Naikhonchhari, Bandarban	92°13′39.84″	21°3′53.84″
S-9	Halidia Para, Naikhonchhari, Bandarban	92°24′19.58″	21°2′27.1″
S-10	Paglachhari, Roangchori, Bandarban	92°24′7.43″	22°2′22.28″
S-11	Paglachhari, Roangchori, Bandarban	92°24′57.68″	22°1′27.89″
S-12	Moddhom Para, Roangchori, Bandarban	92°24′58.85″	21°1′21.96″
S-13	Moddhom Para, Roangchori, Bandarban	92°24′22.86″	22°2′44.64″
S-14	Mong Thoaiching Para, Ruma, Bandarban	92°8′22.93149″	22°22′35.79366″
S-15	Mong Thoaiching Para, Ruma, Bandarban	92°8′57.4917″	22°21′21.50105″
S-16	Mong Thoaiching Para, Ruma, Bandarban	92°10′12.67734″	22°30′57.17177″
S-17	Mong Thoaiching Para, Ruma, Bandarban	92°10′5.75284″	22°31′19.02916″
S-18	Mong Thoaiching Para, Ruma, Bandarban	92°19′46.81″	22°9′27.23″

#### Ethical issues

No explicit rules or regulations pertain to the practice of ethnomedicinal research in Bangladesh. Participants in the study had the purpose of the research project explained to them before they gave oral informed consent. Each participant of the study agreed to participate voluntarily. Participants were allowed to discontinue the interviews at any time. Upon completion of the study, all data will be included online at www.ebbd.info and www.mpbd.info.

### Plant collection, identification, and preservation

Voucher specimens were collected through repeated field trips. While noting the information, care was taken to document all kinds of relevant taxonomic characteristics. The identification was done by consulting with an expert: Professor Dr. Shaikh Bokhtear Uddin, Department of Botany, University of Chittagong, Bangladesh, and through several literature sources. The identified plant species were further compared with the “*Dictionary of Plant Names of Bangladesh* (vascular plants)” (Pasha and Uddin, 2013) for justification of correct scientific names and author citations. Voucher specimens were deposited at the Chittagong University Herbarium (CTGUH), Department of Botany, University of Chittagong, Bangladesh.

### Quantitative ethnobotany

#### Informant Consensus Factor (ICF)

Informant Consensus Factor (Logan, 1986; Heinrich et al., 1998) was calculated using the following formula:

$$\begin{array}{l} \text{FIC}=\text{Nur}-\text{Nt}/\left(\text{Nur}-1\right) \end{array}$$

Where, “Nur” refers to the total number of use reports for each disease cluster and “Nt” refers the total number of species used for that cluster. This formula was used to find out the homogeneity in the ethnomedicinal information documented from the traditional informants.

#### Use Value (UV)

According to Phillips et al. (1994), the UV was calculated using the following formula:

$$\begin{array}{l} \text{UV}=\sum /\text{N} \end{array}$$

Where, “U” refers to the number of uses mentioned by the informants for a given species and “N” refers to the total number of informants interviewed. If a plant secures a high UV score that indicates there are many use reports for that plant, while a low score indicates fewer use reports cited by the informants.

#### Frequency of Citation (FC) and Relative Frequency of Citation (RFC)

The FC was calculated as follows:

$$\begin{array}{l} \text{FC}=\left(\text{Number of times a particular species was mentioned}/\right)\\ \left(\text{total number of times that all species were mentioned}\right)\times 100. \end{array}$$

The RFC index (Tardío and Pardo-De-Santayana, 2008) was evaluated by dividing the number of informants who mentioned the use of the species (FC) by the total number of informants participating in the survey (N). The RFC index ranges from “0” when nobody referred to a plant as useful to “1” when all informants referred to a plant as useful. RFC = FC/N.

#### Relative Importance Index (RI)

According to Tardío and Pardo-De-Santayana (2008), this index was calculated with the following equation:

$$\begin{array}{l} \text{R}{\text{I}}_{\text{s}}=\left\{\text{RF}{\text{C}}_{\text{s}\left(\text{max}\right)}+\text{RN}{\text{U}}_{\text{s}\left(\text{max}\right)}\right\}/2 \end{array}$$

Where, RFC_s(max)_ is the relative frequency of citation over the maximum, i.e., it is obtained by dividing FC_s_ by the maximum value in all species of the survey {RFC_s(max)_ = FC_s_/max(FC)}, and RNU_s(max)_ is the relative number of use-categories over the maximum, obtained dividing the number of uses of the species by the maximum value in all species of the survey {RNU_s(max)_ = NU_s_/max(NU)}. The RI index theoretically varies from 0, when nobody mentioned any use of the plant, to 1, when the plant was most frequently mentioned as useful in the maximum number of use categories.

#### Jaccard Index (JI)

This index is used to compare study data with that of other ethnobotanical studies conducted in other parts of Bangladesh as well as other countries in the world, and also among the indigenous communities in the studied areas. The formula to evaluate the JI index (González-Tejero et al., 2008) was:

JI=cx100/a+b-c, where, “a” is the recorded number of species of the study area “A,” “b” is the documented number of species of the area “B” and “c” is the common number of species in both area “A” and “B.” In case of indigenous communities, “a” is the number of species reported by an indigenous community “A,” “b” is the number of species cited by the indigenous community “B” and c is the number of species reported by both “A” and “B.”

## Results

### Demography of informants

A total of 174 informants were interviewed. Out of these, 129 (74%) were male and 45 (26%) were female. As the Marma were the largest community in the study area, a larger number of informants (99) were interviewed from that community, compared to those from the Chak and Tanchayanga communities. The informants were categorized into five different age groups, as documented in Table 2.

**Table 2:** Demographic characteristics of informants.

**Factor**	**Categories**	**Chak community**	**Marma community**	**Tanchayanga community**	**Total no. of persons**	**Percentage (%)**
Sex	Male	25	74	30	129	74
	Female	9	25	11	45	26
Profession	Government employee	0	5	3	8	4.60
	Teacher	1	3	1	5	2.87
	Farmer	14	33	19	66	37.93
	House wife	6	10	5	21	12.07
	Unemployed	9	24	6	39	22.41
	Professional herbalist	5	21	9	35	20.12
Age	<30	0	11	4	15	8.62
	30–40	6	19	5	30	17.24
	40–50	10	25	18	53	30.46
	50–60	11	22	13	46	26.44
	>60	7	16	7	30	17.24

### Documented plant species and their taxonomy

A total of 159 ethnomedicinal species in 132 genera and 62 families were documented among the informants of the three indigenous communities studied. All documented plant species are presented in Supplementary Table 1, detailing their family, voucher number, local name(s), indigenous name(s), plant part(s) used, ailments treated, frequency of distribution, growth form, source, origin, ethnomedicinal uses, UR, UV, FC, RFC, and RI. Of all plants listed, 128 plants were native and 31 were exotic. In the present study, 129 species were harvested from the wild environment, and 30 plants were cultivated. This study thus highlights the dependence of traditional healers of these three communities in obtaining their ethnomedicines from the natural environment.

Most of the documented species were herbs (53.46%), followed by shrubs (20.13%), trees (18.87%), and climbers (7.55%). Similar results were reported with analogous studies conducted elsewhere (Ghorbani et al., 2011; Singh et al., 2014; Kayani et al., 2015; Malla et al., 2015). The reason for a dominance of herbaceous plant in use is due to the study areas being located in the dense forest zone and herbs being abundantly distributed throughout the study area. The traditional healers preferred to use herbs than other sources, due to comparative ease of collection from deep forest areas, more facile preparation of ethnomedicines and to also enable conservation of the required plant around domestic quarters, churches and pagodas for further use.

**Figure 2:**
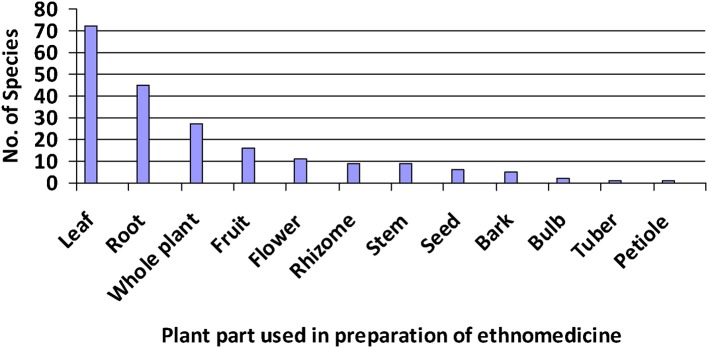
List of plant parts along with their frequency of use among species recorded for the preparation of ethnomedicines.

The most utilized plant parts were leaves (45.28%) followed by roots, whole plants, stems, and so on (Figure 2). Leaves are commonly used for the preparation of herbal medicines due to likely presence of active compounds and comparative ease of phytochemical and pharmacological studies compared to other parts. Ghorbani (2005) noted that leaves are active in food and metabolite production. On the other hand, roots were the second frequently used plant part by healers, likely due to their higher concentration of bioactive compounds than other plant parts (Basualdo et al., 1995).

Dominant families utilized were the Asteraceae (14 species), Lamiaceae (12), Fabaceae (9), Apocynaceae (8), Caesalpiniaceae & Zingiberaceae (7), Rubiaceae & Malvaceae (6), Mimosaceae & Solanaceae (5). Other families were represented by between one and three species. Similar results were reported by other ethnobotanists (Ghorbani et al., 2011; Bibi et al., 2014; Islam et al., 2014; Singh et al., 2014; Fortini et al., 2016; Sadat-Hosseini et al., 2017) while Aston Philander (2011) and Güzel et al. (2015) reported that the Asteraceae was the second largest family in their studies. Our results were also compared with a fundamental book of Bangladeshi Flora, published by Pasha and Uddin (2013). According to them, the top five largest families in Bangladesh are the Poaceae, Fabaceae, Orchidaceae, Rubiaceae, and Asteraceae, respectively, while the Lamiaceae ranked as the 9th largest family. The dominance of Asteraceae and Lamiaceae species in treating ailments may be due to their aromatic characteristics (Güzel et al., 2015) and richness in essential oils (Fortini et al., 2016).

For all species, a frequency of distribution was noted, based on local status and IUCN Red List categories (IUCN, 2017). Based on our field study and local reports, 66 species were categorized as occasional, 45 rare, 41 common, and 7 species abundant. According to the IUCN Red List categorization, 8 plant species were of Least Concern, 2 species were lower risk, and one species (*Dalbergia oliveri* Prain) was endangered, while the rest of the species have not been assessed yet.

### Mode of preparation

**Figure 3:**
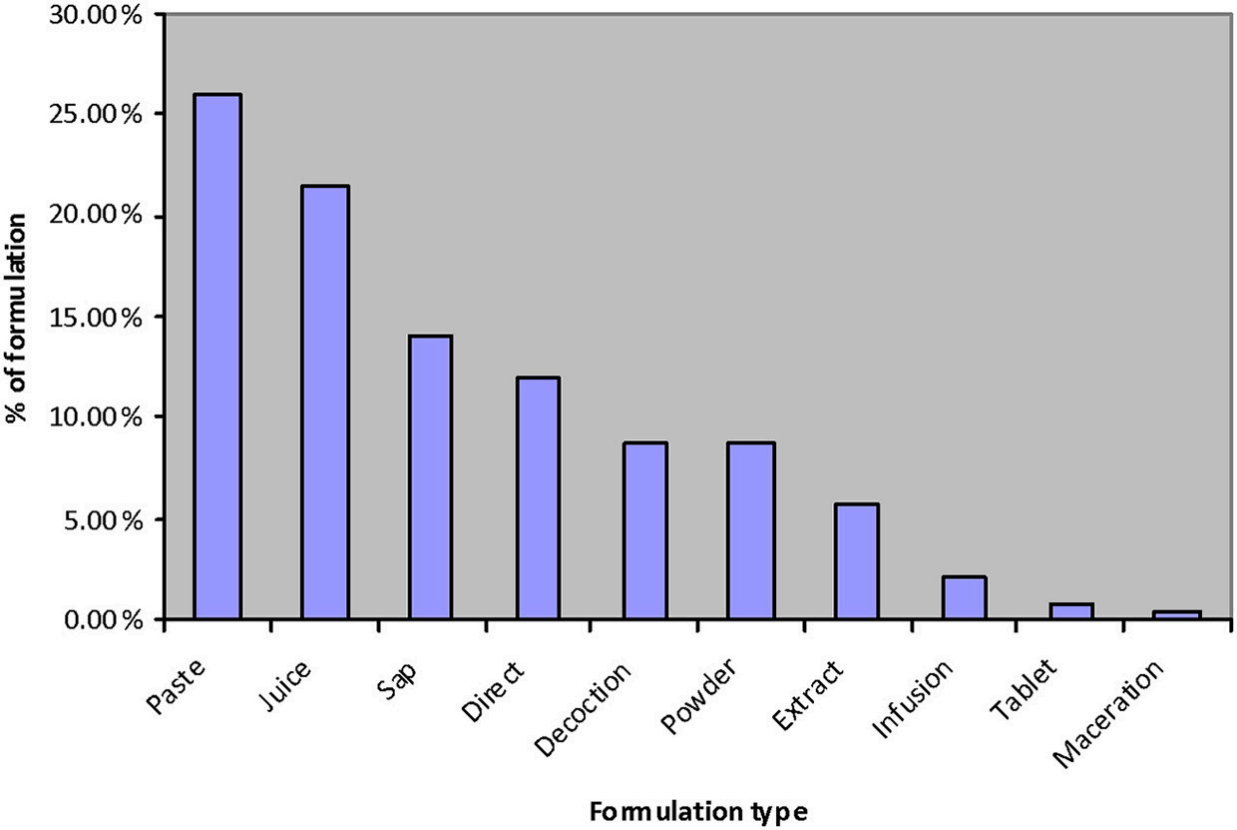
Mode of preparation of ethnomedicines.

The most frequently used mode of preparation was as a paste (63.03%) followed by juices (21.03%), saps (14.05%), direct utilization (11.98%), decoction (8.68%), and so on (Figure 3). Islam et al. (2014) reported that juices were the second highest mode of preparation in their study.

Most of the informants suggested taking herbal medicines orally (75.86%), rather than external (24.11%) use, as consistent with comparable investigations (Kayani et al., 2015; Sadat-Hosseini et al., 2017).

### Quantitative ethnobotany

#### Informant's Consensus Factor (ICF) and species Use Value (UV)

The documented ethnomedicinal plants were used to treat 103 different ailments which were grouped into 17 different categories. The ICF values ranged from 0.65 to 0.77. The highest ICF value of 0.77 was for digestive system disorders followed by parasitic infections (0.76) and treatment of snake and insect bites (0.75), while the lowest ICF value was 0.50 for neurological and psychological disorders (Table 3). Ghorbani et al. (2011) found that digestive system disorders had the highest ICF value, whereas Juárez-Vázquez et al. (2013) noted this as their second highest observed ICF value. This ranking might be due to a lack of adequate knowledge about the pathogenicity of disease and drinking polluted water. As regard to parasitic infections with the second highest ICF value, this is likely due to Bangladesh being one of the 109 countries ranked by the World Health Organization (WHO) as having endemic malaria and the study district being one of the malaria endemic districts of Bangladesh (Haque et al., 2009). The highest number of ethnomedicinal species were used to treat digestive system disorders (40 species) followed by treatment of pain (31) and sexual and related disorders (25), while only two species were documented to treat neurological and psychological disorders (Table 3). Digestive system disorders were those most commonly treated with ethnomedicines in previous studies within Bangladesh (Islam et al., 2014; Rahman et al., 2016) and were also found to be the most common disorders treated in other parts of the world (Hanlidou et al., 2004; Macía et al., 2005; Lee et al., 2008; Aston Philander, 2011; Mati and De Boer, 2011; Suleiman, 2015; Sadat-Hosseini et al., 2017), whereas, de Albuquerque et al. (2007) and Güzel et al. (2015) reported that such disorders were the second most common category treated.

**Table 3:** Informant Consensus Factor (ICF) by category of ailment within the present study.

**S. No**.	**Category of ailment**	**Number of use reports**	**Number of species**	**ICF value**
1	**Digestive system disorders**: Gastritis, diarrhea, ulcers, constipation, digestive aid, piles, carminative, flatulence, indigestion, colic, anthelmintic	174	40	0.77
2	**Parasitic infection**: malaria, liver cyst, scabies	22	6	0.76
3	**Snake, dog and insect bites**	25	7	0.75
4	**Kidney disorders:** kidney and bladder stones, irregular urination, urinary problems, diuretic	20	7	0.68
5	**Fever and cough**	57	19	0.68
6	**Pain:** Abdominal pain, naval pain, toothache, stomachache, earache, breast pain, chest pain, headache, migraine, knee pain, liver pain, sore throat, gout	88	31	0.66
7	**Respiratory disorders:** Asthma, bronchitis, pneumonia	24	9	0.65
8	**General disorders:** beautification of hair and teeth, longevity, multivitamin, dehydration, general weakness, toothpowder, source of calcium, tonic, vomiting, external injuries	27	10	0.65
9	**Microbial infection:** Cholera, dysentery, measles, jaundice, ear infection, fungal infection, chicken pox	25	10	0.62
10	**Rheumatism and fracture:** Rheumatism, bone fracture, paralysis	19	8	0.61
11	**Boils, abscesses, carbuncles, swellings, cuts and wounds**	57	24	0.59
12	**Diabetes, blood circulation and “blood purifiers”**	30	13	0.59
13	**Dermatological:** Allergy, albinism, eczema, ringworm, dandruff, itch, urticaria, cracked heels, baldness, vitiligo	48	21	0.57
14	**Sexual and related disorders:** Dampened sexual desire, excessive bleeding during menstruation and childbirth, enlarged breasts, leucorrhoea, uterine disorders, infertility, spermatorrhea, impotence, abortion, dysmenorrhea.	55	25	0.55
15	**Cancer**	20	10	0.53
16	**Inflammation:** Inflammation, tonsillitis	5	3	0.50
17	**Neurological and psychological disorders:** insanity, analgesic, psychological disorders	3	2	0.50

In the present study, the UV (Supplementary Table 1) ranged between 0.03 and 0.43. Based on UV data, the five most commonly used ethnomedicinal plant species were *Duabanga grandiflora* (0.43), *Zingiber officinale* (0.41), *Congea tomentosa* (0.40), *Matricaria chamomilla* (0.33), and *Engelhardtia spicata* (0.28). The least used species were *Senna alata* and *Senna hirsuta* (0.03 each). These species were used for diverse purposes, including to treat colic, as a sedative, for anti-tumor, anti-allergic, or carminative activity, and to relieve flatulence, gastritis, abdominal pain, coughs and colds, boils and skin disease, while the two species with the lowest UV (*S. alata* and *S. hirsuta*) were solely used to treat eczema and dandruff respectively. Aspects of these results correlate with previous work; Islam et al. (2014) carried out an ethnobotanical survey in another region of Bangladesh and reported *Z. officinale* as having the highest UV in their study, but in the present study it had the second highest UV. Fortini et al. (2016) recorded *M. chamomilla* as having their third highest UV, and the present study recorded this at the fourth highest position.

#### Relative Frequency of Citation (RFC) and Relative Importance Index (RI)

In the present study, RFC values ranged from 0.02 to 0.25. The highest RFC was recorded for *Rauvolfia serpentina* (0.25), followed by *Mimosa pudica* (0.22) and *Scoparia dulcis* (0.20; Supplementary Table 1). The ethnomedicinal plants species having high RFC values indicated their abundant use and widespread knowledge among the local communities. *Rauvolfia serpentina* had the highest frequency of citation (FC-43) but it is a rare species in the study area; thus traditional healers collected this species from the wild and cultivated it adjacent to homes, churches, and pagodas, not only for ethnomedicinal use but also for conservation purposes. Conversely, *M. pudica* (FC-39) and *S. dulcis* (FC-35) were abundantly distributed in the study areas.

The highest RI values were calculated for *S. dulcis* and *Leucas aspera* (0.83 each) followed by *Ricinus communis* (0.76) and *Azadirachta indica* (0.72), while the lowest values were for *Cymbopogon flexuosus* and *Helminthostachys zeylanica* (0.12 each) (Supplementary Table 1).

#### Jaccard Index

A comparison with data reported by ethnobotanists from other regions of Bangladesh as well as internationally was performed by using the Jaccard Index. The original application information of ethnomedicinal plants within our study was compared with 30 previous ethnobotanical research studies published from different countries, including Bangladesh. The JI ranged from 0.32 to 23.24. The top three highest degree of similarities was recorded from Bangladesh with studies conducted by Uddin et al. (2013) with a JI of 23.24, followed by Faruque and Uddin (2014) with a JI of 18.75 and Rahman et al. (2016) with a JI of 18.07 (Table 4). Among neighboring countries, the highest degree of similarity was recorded from India with a JI of 13.30. In Arabic regions, the highest JI (2.69) was found in Turkey. In Africa, the highest JI (2.49) was found in Ethiopia, and in North America, the highest JI (1.87) was recorded in Mexico. The lowest degree of similarity was found with European countries –Portugal and Spain having JIs of 0.32 and 0.33 respectively. Higher similarities in neighboring regions may reflect common flora and similar cultural norms. This is exemplified by India, which shares a 4096 km international border with Bangladesh, the fifth longest such border in the world. Likewise, a lower JI observed from European countries likely reflects the long distance, dissimilar flora, and different cultures between sites.

**Table 4:** Comparison between present and previous studies at neighboring, regional, and global level as performed by Jaccard Index (JI).

**S. N**.	**Previous study area**	**References**	**Total documented species**	**Total documented species in present study**	**Similarl use of plant**	**Dissimilar use of plant**	**Plants common to both areas**	**Jaccard Index (JI)**
1	Arrabida Natural Park, Portugal	Novais et al., 2004	156	159	1	0	1	0.32
2	Pallars, Spain	Agelet and Vallès, 2003	437	159	1	1	2	0.33
3	Northwest Region, Colombia	Otero et al., 2000	101	159	0	1	1	0.39
4	USA	Slish et al., 1999	31	159	1	0	1	0.53
5	Middle Navarra, Spain	Cavero et al., 2011	198	159	2	0	2	0.60
6	Mainarde mountains, Italy	Fortini et al., 2016	106	159	2	0	2	0.77
7	Eastern Mallorca, Spain	Carrió and Vallès, 2012	121	159	1	2	3	0.81
8	South Kerman, Iran	Sadat-Hosseini et al., 2017	115	159	1	2	3	1.12
9	USA	Aston Philander, 2011	205	159	2	2	4	1.14
10	Balochistan, Pakistan	Bibi et al., 2014	102	159	1	3	4	1.54
11	Qaysari Market, Kurdish, Iraq	Mati and De Boer, 2011	82	159	1	3	4	1.66
12	Mato Grosso, Brazil	Ribeiro et al., 2017	309	159	6	2	8	1.80
13	Xalpatlahuac, Mexico	Juárez-Vázquez et al., 2013	67	159	2	2	4	1.87
14	Northern Kordofan region, Sudan	Suleiman, 2015	44	159	3	1	4	2.09
15	Odisha, India	Nagendrappa et al., 2013	16	159	2	2	4	2.45
16	Ethiopia	Teklehaymanot and Giday, 2010	57	159	2	3	5	2.49
17	Hatay Province, Turkey	Güzel et al., 2015	202	159	4	5	9	2.69
18	Uttarakhand, India	Singh et al., 2014	89	159	6	1	7	3.08
19	Yunnan, China	Ghorbani et al., 2011	199	159	7	5	13	4.08
20	Parbat district of Nepal	Malla et al., 2015	132	159	8	3	11	4.26
21	Assam, India	Saikia et al., 2006	85	159	6	10	16	8.16
22	Rangamati, Bangladesh	Uddin et al., 2014	50	159	5	14	19	12.5
23	Uttara Kannada district, India	Bhandary et al., 1995	69	159	11	10	21	12.73
24	Hazarikhil, Chittagong, Bangladesh	Faruque and Uddin, 2011	43	159	6	13	19	13.10
25	Uttar Pradesh, India	Singh et al., 2002	125	159	19	8	27	13.30
26	Atwari, Panchagarh, Bangladesh	Rahman et al., 2016	97	159	8	22	30	18.07
27	Bandarban, Bangladesh	Faruque and Uddin, 2014	66	159	9	18	27	18.75
28	Cox's Bazar, Bangladesh	Uddin et al., 2013	82	159	18	15	33	23.24
29	Alpine & sub-alpine region, Pakistan	Kayani et al., 2015	125	159	1	0	1	0.36
30	Madhupur forest area, Bangladesh	Islam et al., 2014	78	159	15	12	27	17.31

We also calculated the degree of similarity among the three indigenous communities of the study area using the Jaccard Index. A total of seven out of 159 plant species were found to be used by these three indigenous communities with 5 species shared by the Chak and Tanchayanga communities. The degree of similarity found between the Chak and Tanchayanga communities was reflected in a JI of 5.95, followed by Marma and Tanchayanga (JI = 2.34), and Marma and Chak communities (JI = 1.42) (Table 5). It may appear quite surprising that in such similar geographical areas that the overlap of used species is so low, but their treatment systems, cultures, languages, and social structures are distinct. Generally, traditional knowledge was not shared with other communities and it is only transferred to their own generations.

**Table 5:** A comparative study among the three indigenous communities.

**Documented species from Marma**	**Documented species from Chak**	**Documented species from Tanchayanga**	**Common species among Marma, Tanchayanga and Chak**	**Common species between Marma and Chak**	**Common species between Marma and Tanchayanga**	**Common species between Tanchayanga and Chak**	**Jaccard Index (JI) for Marma and Chak**	**JI for Marma & Tanchayanga**	**JI for Tanchayanga and Chak**
92	52	42	7 *Namely-Duabanga grandiflora, Congea tomentosa, Engelhardtia spicata, Chromolaena odorata, Zingiber officinale, Curcuma longa, and Plumeria rubra*	2 *Namely-Ziziphus mauritiana and Hippochaete debilis*	3 *Namely-Alpinia conchigera, Clerodendrum indicum, and Passiflora foetida*	5 *Namely-Aegle marmelos, Equisetum ramosissimum, Asarum cordifolium, Sansevieria trifasciata, and Hemidesmus indicus*	1.42	2.34	5.95

## Discussion

The informants utilized in this study predominantly ranged from 40 to 50 years old (30%), with 44% of the remainder aged 50 or more. This reflects the older profile of the knowledge repository in this community regarding medicinal plant use. With regard to the actual plant materials more commonly used by the people of the Bandarban as assessed by our research, the highest use reports were generated for *R. communis* (7), *A. indica, L. aspera, S. dulcis* (6 each), and *Clitoria ternatea* and *Z. officinale* (5 each). These plants were also reported by other researchers for treating other various disorders in Bangladesh. *Azadirachta indica* is used in eczema and allergy (Khan et al., 2015); chicken pox and measles (Faruque and Uddin, 2014); high blood pressure, gastritis, flatulence, and jaundice (Uddin et al., 2013); pain, wounds small pox, and cough (Islam et al., 2014). *Leucas aspera* is used to treat skin disease (Rahman et al., 2016). *Ricinus communis* is also used for gastritis, diarrhea and dysentery (Islam et al., 2014). *Scoparia dulcis* is used for fever (Khan et al., 2015). *Zingiber officinale* is also used to relief from sore throat (Faruque and Uddin, 2014) and vomiting (Islam et al., 2014).

This survey also reported that many of the documented plants are prescribed for use in combinations. A total of 59 mixtures of medicinal plants and other known or unknown ingredients were recorded. Most commonly, such mixtures included honey (14), seeds of *Nigella sativa* (6), rice-washed water or cow's milk (5 each), or salt and sugar (4 each). In 10 cases, the other ingredients were unknown. The diversity of other ingredients included sparrow birds, crabs, oil, chicken fat, lime, and plants including *Achyranthes aspera, Allium sativum, Averrhoa bilimbi, A. indica, Citrus aurantiifolia, Musa sapientum, Phaseolus vulgaris, Tamarindus indica*, and *Z. officinale*. Most of the mixtures of medicinal plants are used to treat gastrointestinal disorders. The general belief is that such mixtures might enhance the pharmacological activities of medicinal plants (Juárez-Vázquez et al., 2013).

The documented ethnomedicinal information was compared with previous published ethnobotanical studies in the area and with published articles in the databases of SCOPUS, PubMed, BioMed Central, Google Scholar, and Web of Science. The results showed that 16 out of the 159 kinds of species reported in this study reflect newly described therapeutic uses. These species are: *Adiantum capillus-veneris, Agastache urticifolia, Asarum cordifolium, Codariocalyx motorius, C. tomentosa, Curcuma caesia, D. oliveri, E. spicata, Hypserpa nitida, Jacquemontia paniculata, Leucas zeylanica, Maesa indica, Merremia vitifolia, Scutellaria discolor, Smilax odoratissima*, and *Torenia asiatica* (see uses in Supplementary Table 1). Interestingly, seven of these species have not been pharmacologically studied to date. These are: *Agastache urticifolia, Asarum cordifolium, C. tomentosa, E. spicata, Hypserpa nitida, Merremia vitifolia*, and *Smilax odoratissima*. Future work is necessary to investigate the pharmacological properties of these plants species, in order to validate their traditional use. Furthermore, two ethnomedicinal species (*C. tomentosa* and *E. spicata*), with third and fifth highest use values respectively, are used to treat tumors and breast cancer by three indigenous communities; therefore, these species warrant particular pharmacological investigation.

## Conclusion

The present study showed that traditional treatment systems using medicinal plants is still prevalent in the studied areas, and it underlines the importance in the documentation of traditional ethnomedicinal knowledge before losing this diverse resource. To the best of our knowledge, this is the first quantitative ethnomedicinal study in the study area indicating UV, ICF, FC, RFC, RI, and JI indices. The present study records new ethnomedicinal species with their therapeutic uses, which can potentially lead to the development of new therapies and may represent novel bioresources for phytochemical and pharmacological studies, notably *C. tomentosa* and *E. spicata*, which have claimed anticancer effects by the healers of all studied indigenous communities in the study area.

## Ethics statement

The study was carried out in accordance with the recommendations of the Code of Ethics of the International Society of Ethnobiology. Ethics approval was not required by the university of the principal author. Verbal informed consent was obtained from each informant prior to all interviews. During this discussion, the research objectives, interview procedure were explained to each informant and confidentiality was assured. Consent for audio recording was also obtained.

## Author contributions

Designed the study: MF and XH; Data Collection: MF and SU; Analyzed the data: MF and XL; Wrote the manuscript: MF, XH, JB, and SU. All authors read and approved the final manuscript.

### Conflict of interest statement

The authors declare that the research was conducted in the absence of any commercial or financial relationships that could be construed as a potential conflict of interest.

## References

[B1] Agelet A. Vallès J. (2003). Studies on pharmaceutical ethnobotany in the region of Pallars (Pyrenees, Catalonia, Iberian Peninsula). Part II. New or very rare uses of previously known medicinal plants. J. Ethnopharmacol. 84, 211–227. 10.1016/S0378-8741(02)00319-7 12648818

[B2] Alexiades M. N. Sheldon J. W. (1996). Selected Guidelines for Ethnobotanical Research: A Field Manual. New York, NY: New York Botanical Garden.

[B3] Asase A. Kokubun T. Grayer R. J. Kite G. Simmonds M. S. Oteng-Yeboah A. A.. (2008). Chemical constituents and antimicrobial activity of medicinal plants from Ghana: *Cassia sieberiana, Haematostaphis barteri, Mitragyna inermis* and *Pseudocedrela kotschyi*. Phytother. Res. 22, 1013–1016. 10.1002/ptr.2392 18618525

[B4] Aston Philander L. (2011). An ethnobotany of Western Cape Rasta bush medicine. J. Ethnopharmacol. 138, 578–594. 10.1016/j.jep.2011.10.004 22004893

[B5] Banglapedia (2003). National Encyclopedia of Bangladesh. Dhaka: Asiatic Society of Bangladesh.

[B6] Basualdo I. Zardini E. M. Ortiz M. (1995). Medicinal plants of Paraguay: underground Organs, II. Econ. Bot. 49, 387–394. 10.1007/BF02863089

[B7] Bhandary M. J. Chandrashekar K. R. Kaveriappa K. M. (1995). Medical ethnobotany of the Siddis of Uttara Kannada district, Karnataka, India. J. Ethnopharmacol. 47, 149–158. 10.1016/0378-8741(95)01274-H 8569239

[B8] Bibi T. Ahmad M. Bakhsh Tareen R. Mohammad Tareen N. Jabeen R. Rehman S. U.. (2014). Ethnobotany of medicinal plants in district Mastung of Balochistan province-Pakistan. J. Ethnopharmacol. 157, 79–89. 10.1016/j.jep.2014.08.042 25260579

[B9] Bibi T. Ahmad M. Mohammad Tareen N. Jabeen R. Sultana S. Zafar M.. (2015). The endemic medicinal plants of Northern Balochistan, Pakistan and their uses in traditional medicine. J. Ethnopharmacol. 173, 1–10. 10.1016/j.jep.2015.06.050 26187276

[B10] Carrió E. Vallès J. (2012). Ethnobotany of medicinal plants used in Eastern Mallorca (Balearic Islands, Mediterranean Sea). J. Ethnopharmacol. 141, 1021–1040. 10.1016/j.jep.2012.03.049 22783553

[B11] Cavero R. Y. Akerreta S. Calvo M. I. (2011). Pharmaceutical ethnobotany in the Middle Navarra (Iberian Peninsula). J. Ethnopharmacol. 137, 844–855. 10.1016/j.jep.2011.07.001 21767624

[B12] de Albuquerque U. P. Hanazaki N. (2009). Five problems in current ethnobotanical research—and some suggestions for strengthening them. Hum. Ecol. 37, 653–661. 10.1007/s10745-009-9259-9

[B13] de Albuquerque U. P. Monteiro J. M. Ramos M. A. De Amorim E. L. (2007). Medicinal and magic plants from a public market in northeastern Brazil. J. Ethnopharmacol. 110, 76–91. 10.1016/j.jep.2006.09.010 17056216

[B14] Fabricant D. S. Farnsworth N. R. (2001). The value of plants used in traditional medicine for drug discovery. Environ. Health Perspect. 109:69. 10.1289/ehp.01109s169 11250806 PMC1240543

[B15] Faruque M. Uddin S. (2014). Ethnomedicinal study of the Marma community of Bandarban district of Bangladesh. Acad. J. Med. Plants 2, 014–025. 10.15413/ajmp.2013.0140

[B16] Faruque O. Uddin S. B. (2011). Ethnodiversity of medicinal plants used by Tripura community of Hazarikhil in Chittagong district of Bangladesh. J. Taxon. Biodiv. Res. 5, 27–32. Available online at: http://www.jtbrbd.org/volume-5/volume-5-paper-6.pdf

[B17] Fortini P. Di Marzio P. Guarrera P. M. Iorizzi M. (2016). Ethnobotanical study on the medicinal plants in the Mainarde Mountains (central-southern Apennine, Italy). J. Ethnopharmacol. 184, 208–218. 10.1016/j.jep.2016.03.010 26969402

[B18] Ghorbani A. (2005). Studies on pharmaceutical ethnobotany in the region of Turkmen Sahra, north of Iran: (Part 1): general results. J. Ethnopharmacol. 102, 58–68. 10.1016/j.jep.2005.05.035 16024194

[B19] Ghorbani A. Langenberger G. Feng L. Sauerborn J. (2011). Ethnobotanical study of medicinal plants utilised by Hani ethnicity in Naban River Watershed National Nature Reserve, Yunnan, China. J. Ethnopharmacol. 134, 651–667. 10.1016/j.jep.2011.01.011 21251966

[B20] González-Tejero M. R. Casares-Porcel M. Sánchez-Rojas C. P. Ramiro-Gutiérrez J. M. Molero-Mesa J. Pieroni A.. (2008). Medicinal plants in the Mediterranean area: synthesis of the results of the project Rubia. J. Ethnopharmacol. 116, 341–357. 10.1016/j.jep.2007.11.045 18242025

[B21] Güzel Y. Güzelşemme M. Miski M. (2015). Ethnobotany of medicinal plants used in Antakya: a multicultural district in Hatay Province of Turkey. J. Ethnopharmacol. 174, 118–152. 10.1016/j.jep.2015.07.042 26239155

[B22] Hanlidou E. Karousou R. Kleftoyanni V. Kokkini S. (2004). The herbal market of Thessaloniki (N Greece) and its relation to the ethnobotanical tradition. J. Ethnopharmacol. 91, 281–299. 10.1016/j.jep.2004.01.007 15120452

[B23] Haque U. Ahmed S. M. Hossain S. Huda M. Hossain A. Alam M. S. (2009). Malaria prevalence in endemic districts of bangladesh. PLoS ONE 4:e6737. 10.1371/journal.pone.0006737 19707580 PMC2726938

[B24] Heinrich M. (2000). Ethnobotany and its role in drug development. Phytother. Res. 14, 479–488. 10.1002/1099-1573(200011)14:7<479::AID-PTR958>3.0.CO;2-2 11054835

[B25] Heinrich M. Ankli A. Frei B. Weimann C. Sticher O. (1998). Medicinal plants in Mexico: healers' consensus and cultural importance. Soc. Sci. Med. 47, 1859–1871. 10.1016/S0277-9536(98)00181-6 9877354

[B26] Heinrich M. Bremner P. (2006). Ethnobotany and ethnopharmacy-their role for anti-cancer drug development. Curr. Drug Targets 7, 239–245. 10.2174/138945006776054988 16515525

[B27] Islam M. K. Saha S. Mahmud I. Mohamad K. Awang K. Jamal Uddin S.. (2014). An ethnobotanical study of medicinal plants used by tribal and native people of Madhupur forest area, Bangladesh. J. Ethnopharmacol. 151, 921–930. 10.1016/j.jep.2013.11.056 24342778

[B28] Islam R. (2006). Role of plant medicine in health care and improving nutritional standard in rural area of Bangladesh, in National Seminar on Diversity of Medicinal Plants and Their Sustainable Utilization in Health Care and Improving Nutritional Standard in Rural Areas (Kolkata), 1–30.

[B29] IUCN (2017). The IUCN Red List of Threatened Species. Available online at: http://www.iucnredlist.org

[B30] Juárez-Vázquez M. D. C. Carranza-Álvarez C. Alonso-Castro A. J. González-Alcaraz V. F. Bravo-Acevedo E. Chamarro-Tinajero F. J.. (2013). Ethnobotany of medicinal plants used in Xalpatlahuac, Guerrero, México. J. Ethnopharmacol. 148, 521–527. 10.1016/j.jep.2013.04.048 23665055

[B31] Kabir M. H. Hasan N. Rahman M. M. Rahman M. A. Khan J. A. Hoque N. T.. (2014). A survey of medicinal plants used by the Deb barma clan of the Tripura tribe of Moulvibazar district, Bangladesh. J. Ethnobiol. Ethnomed. 10:19. 10.1186/1746-4269-10-19 24502444 PMC3996145

[B32] Kadir M. F. Sayeed M. S. B. Mia M. M. (2013). Ethnopharmacological survey of medicinal plants used by traditional healers in Bangladesh for gastrointestinal disorders. J. Ethnopharmacol. 147, 148–156. 10.1016/j.jep.2013.02.023 23458917

[B33] Kayani S. Ahmad M. Sultana S. Khan Shinwari Z. Zafar M. Yaseen G.. (2015). Ethnobotany of medicinal plants among the communities of Alpine and Sub-alpine regions of Pakistan. J. Ethnopharmacol. 164, 186–202. 10.1016/j.jep.2015.02.004 25680839

[B34] Khan M. A. Islam M. K. Siraj M. A. Saha S. Barman A. K. Awang K.. (2015). Ethnomedicinal survey of various communities residing in Garo Hills of Durgapur, Bangladesh. J. Ethnobiol. Ethnomed. 11, 44. 10.1186/s13002-015-0033-3 26025456 PMC4488057

[B35] Lee S. Xiao C. Pei S. (2008). Ethnobotanical survey of medicinal plants at periodic markets of Honghe Prefecture in Yunnan Province, SW China. J. Ethnopharmacol. 117, 362–377. 10.1016/j.jep.2008.02.001 18359178

[B36] Logan M. H. (1986). Informant consensus: a new approach for identifying potentially effective medicinal plants, in Plants in Indigenous Medicine and Diet: Biobehavioral Approaches, ed Etkin N. L. (Bedford Hills, NY: Redgrave publishers), 91–112.

[B37] Macía M. J. García E. Vidaurre P. J. (2005). An ethnobotanical survey of medicinal plants commercialized in the markets of La Paz and El Alto, Bolivia. J. Ethnopharmacol. 97, 337–350. 10.1016/j.jep.2004.11.022 15707774

[B38] Malla B. Gauchan D. P. Chhetri R. B. (2015). An ethnobotanical study of medicinal plants used by ethnic people in Parbat district of western Nepal. J. Ethnopharmacol. 165, 103–117. 10.1016/j.jep.2014.12.057 25571849

[B39] Martin G. J. (1995). Ethnobotany: A Methods Manual. London, UK: Earthscan.

[B40] Mati E. de Boer H. (2011). Ethnobotany and trade of medicinal plants in the Qaysari Market, Kurdish Autonomous Region, Iraq. J. Ethnopharmacol. 133, 490–510. 10.1016/j.jep.2010.10.023 20965241

[B41] Nadembega P. Boussim J. I. Nikiema J. B. Poli F. Antognoni F. (2011). Medicinal plants in Baskoure, Kourittenga Province, Burkina Faso: an ethnobotanical study. J. Ethnopharmacol. 133, 378–395. 10.1016/j.jep.2010.10.010 20950680

[B42] Nagendrappa P. B. Naik M. P. Payyappallimana U. (2013). Ethnobotanical survey of malaria prophylactic remedies in Odisha, India. J. Ethnopharmacol. 146, 768–772. 10.1016/j.jep.2013.02.003 23434608

[B43] Novais M. H. Santos I. Mendes S. Pinto-Gomes C. (2004). Studies on pharmaceutical ethnobotany in Arrabida Natural Park (Portugal). J. Ethnopharmacol. 93, 183–195. 10.1016/j.jep.2004.02.015 15234752

[B44] Otero R. Fonnegra R. Jiménez S. L. Núñez V. Evans N. Alzate S. P.. (2000). Snakebites and ethnobotany in the northwest region of Colombia: Part I: traditional use of plants. J. Ethnopharmacol. 71, 493–504. 10.1016/S0378-8741(00)00243-9 10940589

[B45] Pasha M. Uddin S. (2013). Dictionary of Plant Names of Bangladesh (Vascular plants). Chittagong: Janokalyan Prokashani.

[B46] Phillips O. Gentry A. H. Reynel C. Wilkin P. Galvez-Durand B. C. (1994). Quantitative ethnobotany and amazonian conservation etnobotánica cuantitativa y la conservación de la amazonia. Conserv. Biol. 8, 225–248.

[B47] Rahman K. R. Faruque M. O. Uddin S. B. Hossen I. (2016). Ethnomedicinal knowledge among the local community of Atwari Upazilla of Panchagarh District, Bangladesh. Int. J. Trop. Agric. 34, 1323–1335. Available online at: http://serialsjournals.com/serialjournalmanager/pdf/1484128065.pdf

[B48] Ribeiro R. V. Bieski I. G. C. Balogun S. O. Martins D. T. O. (2017). Ethnobotanical study of medicinal plants used by Ribeirinhos in the North Araguaia microregion, Mato Grosso, Brazil. J. Ethnopharmacol. 205, 69–102. 10.1016/j.jep.2017.04.023 28476677

[B49] Sadat-Hosseini M. Farajpour M. Boroomand N. Solaimani-Sardou F. (2017). Ethnopharmacological studies of indigenous medicinal plants in the south of Kerman, Iran. J. Ethnopharmacol. 199, 194–204. 10.1016/j.jep.2017.02.006 28167292

[B50] Saikia A. P. Ryakala V. K. Sharma P. Goswami P. Bora U. (2006). Ethnobotany of medicinal plants used by Assamese people for various skin ailments and cosmetics. J. Ethnopharmacol. 106, 149–157. 10.1016/j.jep.2005.11.033 16473486

[B51] Singh A. K. Raghubanshi A. S. Singh J. S. (2002). Medical ethnobotany of the tribals of Sonaghati of Sonbhadra district, Uttar Pradesh, India. J. Ethnopharmacol. 81, 31–41. 10.1016/S0378-8741(02)00028-4 12020925

[B52] Singh H. Husain T. Agnihotri P. Pande P. C. Khatoon S. (2014). An ethnobotanical study of medicinal plants used in sacred groves of Kumaon Himalaya, Uttarakhand, India. J. Ethnopharmacol. 154, 98–108. 10.1016/j.jep.2014.03.026 24685588

[B53] Slish D. F. Ueda H. Arvigo R. Balick M. J. (1999). Ethnobotany in the search for vasoactive herbal medicines. J. Ethnopharmacol. 66, 159–165. 10.1016/S0378-8741(98)00225-6 10433472

[B54] Suleiman M. H. (2015). An ethnobotanical survey of medicinal plants used by communities of Northern Kordofan region, Sudan. J. Ethnopharmacol. 176, 232–242. 10.1016/j.jep.2015.10.039 26519203

[B55] Tardío J. Pardo-De-Santayana M. (2008). Cultural importance indices: a comparative analysis based on the useful wild plants of Southern Cantabria (Northern Spain)1. Econ. Bot. 62, 24–39. 10.1007/s12231-007-9004-5

[B56] Teklehaymanot T. Giday M. (2010). Quantitative ethnobotany of medicinal plants used by Kara and Kwego semi-pastoralist people in lower Omo River Valley, Debub Omo Zone, Southern Nations, Nationalities and Peoples Regional State, Ethiopia. J. Ethnopharmacol. 130, 76–84. 10.1016/j.jep.2010.04.013 20420888

[B57] Uddin S. B. (2014). Bangladesh Ethnobotany Online Database. Available online at: www.ebbd.info

[B58] Uddin S. B. Faruque M. O. Talukder S. (2014). A Survey of Traditional Health Remedies of the Chakma Indigenous Community of Rangamati District, Bangladesh. Plant Sci. Res. 1:106.

[B59] Uddin S. B. Ratna R. S. Faruque M. O. (2013). Ethnobotanical Study on Medicinal Plants of Rakhaing Indigenous Community of Cox's Bazar District of Bangladesh. J. Pharmacogn. Phytochem. 2, 164–174. Available online at: http://www.phytojournal.com/archives/?year=2013&vol=2&issue=4&part=C&ArticleId=226

[B60] Van Wyk B.-E. Oudtshoorn B. V. Gericke N. (1997). Medicinal Plants of South Africa. Johannesburg: Briza.

[B61] Yusuf M. Begum J. Hoque M. N. Chowdhury J. U. (2009). Medicinal Plants of Bangladesh. Dhaka: Bangladesh Council of Scientific and Industrial Research.

