# Supplementary material for: Quantitative Ethnobotany of Medicinal Plants Used by Indigenous Communities in the Bandarban District of Bangladesh
Source: Front Pharmacol. 2018 Feb 6;9:40. doi: 10.3389/fphar.2018.00040 (PMC5808248; doi:10.3389/fphar.2018.00040)
Supplement: Supplementary file 1 [file Table1.doc]

Supplementary Table 1: List of ethnomedicinal plant species from the study area

| **Scientific name and voucher number & IUCN conservation status** | **Family in nomenclature** | **Local name** | **Pangkhua/Chak/Marma name** | **Origin** | **Growth form** | **Part(s) used** | **Source** | **Disease/Ailments treated** | **Previous reports** | **Ethnomedicinal Uses** | **F o D** | **UR** | **UV** | **FC** | **RFC** | **RI** |
| --- | --- | --- | --- | --- | --- | --- | --- | --- | --- | --- | --- | --- | --- | --- | --- | --- |
| *Abroma augusta* (L.) L.f.; MCT-F-139 | Malvaceae | Ulatkambol | Agunitita/T | N | S | R | W | *Stomachache, Excessive bleeding after delivery* | 28●,  26♦,30♦ | Sap of the root is taken at a dose of two spoonfuls twice daily for three days to treat stomachache. Burned root is rubbed on stone and the resultant sap is taken with sugar once daily following excessive bleeding after delivery. | Rr | 2 | 0.25 | 8 | 0.04 | 0.24 |
| *Acacia concinna* (Wild.) DC.; MCT-F-117 | Mimosaceae | Banritha | Unknown/M | N | T | R | W | *Menstrual problems, Irregular urination* |  | Juice of the root is taken for three months to treat menstrual problems. Sap of the root with sugar is taken to treat irregular urination. | O | 2 | 0.10 | 19 | 0.11 | 0.36 |
| *Acacia pennata* (L.) Willd.; MCT-F-027 | Mimosaceae | Aila | Rupoi/M | E | T | R | W | *Bone fracture* |  | Paste of root is applied to bone fractures. | O | 1 | 0.08 | 12 | 0.07 | 0.21 |
| *Achyranthes aspera* L.  MCT-F-004 | Amaranthaceae | Apang | Chai-ka-killung/M | N | H | R, WP | W | *Abortion, allergy; toothache* | 22●, 26●,27●,16♦, 22♦,25♦ | Sap of root is taken to induce abortion. The powdered whole plant, combined with coconut oil, is applied to affected areas in skin allergy. The root is directly chewed in toothache. | Cm | 3 | 0.14 | 21 | 0.12 | 0.46 |
| *Acmella radicans*  [(Jacq.) R.K.Jansen](http://www.theplantlist.org/tpl1.1/record/gcc-34111); MCT-F-022 | Asteraceae | Unknown | Unknown/C | N | H | WP | W | *Boils, Pneumonia* |  | A paste of the whole plant is applied to boils while the juice of the whole plant is taken at a dose of two teaspoonfuls twice daily to treat in pneumonia. | C | 2 | 0.18 | 11 | 0.06 | 0.27 |
| *Acorus calamus* L.; MCT-F-096  Least Concern | Acoraceae | Botch | Laingyu/C | N | H | L | C | *Indigestion* | 19●,30●, 9♦,  22♦,26♦,27♦,28♦ | Juice of the fresh leaves combined with honey is given at a dose of two teaspoonfuls twice daily for three days after food to treat indigestion | Rr | 1 | 0.20 | 5 | 0.03 | 0.13 |
| *Adiantum capillus-veneris* L. ; MCT-F-110  Least Concern | Adiantaceae | Venichadda | Sonamukhi pata/M | N | H | WP | W | *Jaundice, Diabetes* |  | A decoction of the whole plant is taken at a dose of one cupful three times daily for ten days to cure jaundice, and the same dose is taken for three months for the treatment of diabetes. | O | 2 | 0.20 | 10 | 0.06 | 0.26 |
| *Aegle marmelos* (L.) Corrêa; MCT-F-051 | Rutaceae | Bel | Uraifang/C; Unknown/T | N | T | Fr | C | *Digestive, dysentery, diarrhea* | 18●,24●,25●,30●, 21♦,27♦,28♦ | Juice of the ripe fruit is taken with honey and salt in dysentery and diarrhea (/T and /C). The powdered skin of green fruit is taken as a digestive (/T). | O | 3 | 0.12 | 25 | 0.14 | 0.50 |
| *Agastache urticifolia*  (Benth.) Kuntze; MCT-F-047 | Lamiaceae | Unknown | Unknown/T | E | H | L | W | *Diarrhea* |  | A decoction of leaves is taken at a dose of one cupful three times daily for ten days in cases of diarrhea. | Rr | 1 | 0.17 | 6 | 0.03 | 0.14 |
| *Allium sativum* L.; MCT-F-014 | Liliaceae | Rusun | Chungfro/C | E | H | Bb | C | *Blood circulation, boils* | 2●, 5●, 17♦, 22♦,26♦,27♦,28♦ | The bulb is directly taken with vegetables or fish to maintain normal blood circulation. A paste of the bulb is applied to boils. | Cm | 2 | 0.09 | 23 | 0.13 | 0.41 |
| *Aloe barbadensis* Mill.; MCT-F-078 | Xanthorrhoeaceae | Unknown | Unknown /M | E | H | WP | C | *Fractures of bone* | 21●, 30♦ | Sap of the plant is applied to fractures. | Rr | 1 | 0.07 | 14 | 0.08 | 0.23 |
| *Alpinia conchigera* Griff.; MCT-F-003 | Zingiberaceae | Konchi elachi | Khetranga/T; podogro/M | N | S | R, Rz | C | *Abdominal pain, naval pain, menstrual problems* |  | A tablet prepared from the root alongside other unknown ingredients is taken twice daily for ten days for abdominal and naval pain (/T). The juice of the rhizome is taken at a dose of one cupful twice daily to treat menstrual problems (/M) | O | 3 | 0.14 | 22 | 0.13 | 0.47 |
| *Amaranthus spinosus* L.; MCT-F-095 | Amaranthaceae | Katamaris | Hanuhuya/M | N | H | WP | W | *Increasing sexual desire, vomiting* | 16●, 24●, 25●, 21♦ | The whole plant is cooked along with sparrow birds, and eaten as a curry to increase sexual desire. The juice of the whole plant is taken to stop vomiting. | Cm | 2 | 0.18 | 11 | 0.06 | 0.27 |
| *Anagallis arvensis* L.; MCT-F-113 | Primulaceae | Pakhichosha | Unknown/T | N | H | L | W | *Liver Cysts* |  | A decoction of leaves is given with other ingredients at a dose of one cupful daily to treat liver cysts. | Rr | 1 | 0.17 | 6 | 0.03 | 0.14 |
| *Ananus comosus* (L.) Merr.; MCT-F-043 | Bromeliaceae | Anaros | Chung-omra/m | E | H | L | C | *Dehydration, general weakness* | 28●, 12♦, 30♦ | Young leaves are eaten along with a trace amount of salt and sugar in dehydration and general weakness. | Cm | 2 | 0.09 | 23 | 0.13 | 0.41 |
| *Argemone maxicana* L.; MCT-F-140 | Papaveraceae | Shialkanta | Chakka/M | E | S | R | W | *Stomachache, Gas form* | 13♦, 25♦, 26♦ | Root sap is taken directly to treat stomachache and to reduce gas in the stomach. | O | 2 | 0.13 | 15 | 0.09 | 0.32 |
| *Aristolochia indica* L. MCT-F-025 | Aristochiaceae | Isharmul | Sikhingkouao/M | N | C | Fr | W | *Boils, cough* |  | A paste of the fruit is used to cure boils on the finger. Sap of the fruit is taken twice daily for two days in cases of cough in children. | O | 2 | 0.17 | 12 | 0.07 | 0.28 |
| *Arundo donax* L.; MCT-F-136 | Poaceae | Nol | Keukhagra/m | N | S | R | W | *Sore throat , diabetes* | 6● | Root sap is taken directly to treat sore throat. An infusion of the root is taken in diabetes. | O | 2 | 0.11 | 18 | 0.10 | 0.35 |
| *Asarum cordifolium* C.E.C.Fisch.; MCT-F-065 | Aristolochiaceae | Asrum | Unknown/T; /C | N | H | R | W | *Post-partum breast enlargement ,breast pain* |  | The breast is washed with root sap along with rice-washed water to relieve pain (/T), and is also taken orally at a dose of two teaspoonfuls twice daily for seven days to prevent breast enlargement following delivery(/C). | Rr | 2 | 0.15 | 13 | 0.07 | 0.29 |
| *Asparagus racemosus* Wild. ; MCT-F-005 | Asparagaceae | Shotomuli | Sattir chara/T | N | H | R | C | *Abortion, rheumatism, spermatorrhoea* |  | Juice of the root is taken as a single dose of three teaspoonfuls on an empty stomach to induce abortion. A paste of the root is applied and massaged slowly to relieve pain in rheumatism. The root extract is taken with honey and black cumin to cure spermatorrhoea. | Rr | 3 | 0.12 | 25 | 0.14 | 0.50 |
| *Averrhoa bilimbi* L.; MCT-F-093 | Oxalidaceae | Bilimbi | Jipru/M | N | T | R | C | *To increase sexual desire* |  | The root sap is taken directly to increase sexual desire. | O | 1 | 0.14 | 7 | 0.04 | 0.15 |
| [*Ayapana* *triplinervis* (Vahl) R.M.King & H.Rob.](http://www.theplantlist.org/tpl1.1/record/gcc-41590); MCT-F-120 | Asteraceae | Ayapan | Crusedung/C | E | H | Fw, L;R | C | *Paralysis, Tonsillitis, abdominal pain* | 18●, 20●, 24●, 28●,9♦, 23♦,25♦, 26♦,30♦ | A paste of the flowers, leaves and root is rubbed on paralyzed areas. Young leaves are taken for the treatment of tonsillitis; the juice of the root is used to treat abdominal pain. | O | 3 | 0.13 | 23 | 0.13 | 0.48 |
| *Azadirachta indica* A.Juss.; MCT-F-103 | Meliaceae | Nim | Toimotahk/C | N | T | B, L, Sd | W | *Insecticide, diabetes, fever, skin diseases, piles, malaria* | 21●,22●,25●,26●,30●,27♦ | Leaves are made into a paste with a small amount of lime and applied externally in skin diseases. Stem bark juice is taken with honey once daily in fever, piles and malaria. Powdered seed is given in diabetes. Leaves are directly used in the living room as insecticide. Powdered leaves are also applied to crop lands as an insecticide. | O | 6 | 0.24 | 25 | 0.14 | 0.72 |
| *Bauhinia purpurea* L.  ; MCT-F-006  Least Concern | Caesalpiniaceae | Debkanchan | Unknown /M | E | T | S,  Fw,L | C | *Albinism, cracked heels* |  | Stem sap is applied to cracked heels. Sap of the stem and flower are applied to affected areas in albinism. | Rr | 2 | 0.12 | 17 | 0.10 | 0.34 |
| *Begonia annulata* K. Koch  MCT-F-044 | Begoniaceae | Gonibata | Chang-chi-mani/M | N | H | P | W | *Cough* |  | The leaf petiole is directly taken to treat cough. | O | 1 | 0.10 | 10 | 0.06 | 0.19 |
| *Blumea balsamifera* (L.) DC.; MCT-F-087 | Asteraceae | Nagor chandal | Gaise ola/T | N | H | L | W | *Headache* | 19● | A paste of the leaves is applied to the forehead to relieve pain. | O | 1 | 0.17 | 6 | 0.03 | 0.14 |
| *Blumea lacera* (Burm.f.) DC.; MCT-F-105 | Asteraceae | Barokukshima | Fao ma/M | N | H | R | W | *Irregular Menstruation, stomachache* | 22●, 27♦ | The root sap is taken along with rice-washed water at a dose of one cupful daily for three days to cure irregular menstruation. The root is rubbed on stone and the resultant sap is taken to treat stomachache. | O | 2 | 0.18 | 11 | 0.06 | 0.27 |
| *Blumea membranacea* DC.; MCT-F-032 | Asteraceae | Patlapata | Crokidongpa/M | N | H | R | W | *Diarrhea in children* |  | The root sap is given at a dose of one teaspoonful twice daily until cure of diarrhea in children | Cm | 1 | 0.10 | 10 | 0.06 | 0.19 |
| *Bridelia stipularis* (L.) Blume; MCT-F-046 | Phyllanthaceae | Harinhara | Paingkhachi/M | N | T | Fr, L | W | *Diarrhea* | 22♦ | Juice of the fruit and leaves is taken (as much as possible) until a cure of diarrhea is observed. | O | 1 | 0.04 | 22 | 0.13 | 0.33 |
| [***Bryophyllum* *pinnatum* (Lam.) Oken**](http://www.theplantlist.org/tpl1.1/record/kew-2684576)**;** MCT-F-038 | Crassulaceae | Kaphpata | Raikkhapompom/M | N | H | L | W | *Cough, kidney stones, cuts and wounds* | 20●,23●,28●,21♦,22♦,26♦,27♦,30♦ | An extract of leaves, along with other ingredients (unknown) is given at a dose of one cupful twice daily until cure of cough. The juice of the leaves is taken at a dose of one teaspoonful twice daily for ten days to remove stones from the kidney. A paste of the leaves is applied directly on cuts and wounds. | O | 3 | 0.09 | 34 | 0.19 | 0.61 |
| *Buddleja asiatica* Lour; MCT-F-070 | Scrophulariaceae | Budbhota | Keu-machui/M | N | S | R | W | *Excessive bleeding during delivery* |  | Juice of the root is taken at a dose of half a tea cupful three times daily for ten days to stop excessive bleeding during delivery. | Cm | 1 | 0.05 | 19 | 0.11 | 0.29 |
| *Bulbophyllum pectinatum*  Finet; MCT-F-059 | Orchidaceae | Bulbopec | Saittalhi/M | N | H | L | W | *Ear infections* | 24♦ | Sap of the leaves is used to treat ear infections. | O | 1 | 0.06 | 16 | 0.09 | 0.26 |
| *Caesalpinia bonducella* (L.) Fleming; MCT-F-015 | Caesalpiniaceae | Nutsela | Maleisabrengsing/C | E | S | Sd | W | *Boils* |  | Powdered seed is used for the treatment of boils. | O | 1 | 0.08 | 13 | 0.07 | 0.22 |
| *Callicarpa arborea* Roxb.; MCT-F-074 | Lamiaceae | Bormala | Tarama/M | N | S | R | W | *Fever, headache* | 19●,20● | Root sap is taken twice daily for three days to cure fever and headache. | O | 2 | 0.11 | 18 | 0.10 | 0.35 |
| *Calotropis gigantea* (L.) Dryand; MCT-F-036 | Apocynaceae | Akand | Maru/M | N | S | B,L,R | W | *Cough, asthma,*  *rheumatism* | 14●,24●,26●,28●,23♦,27♦,30♦ | Juices of bark, leaves and root are taken along with other ingredients to treat cough and asthma. Warm leaves are massaged on the skin to treat rheumatism. | Cm | 3 | 0.12 | 25 | 0.14 | 0.50 |
| *Calotropis procera* **(Aiton) Dryand** MCT-F-056 | Apocynaceae | Swet akand | Unknown /M | N | H | L | W | *Diabetes* |  | Leaves are eaten to treat diabetes. | O | 1 | 0.20 | 5 | 0.03 | 0.13 |
| *Cassia fistula* L.; MCT-F-054 | Caesalpiniaceae | Sonalu | Honalu/C | N | T | L, Fr | W | *Dysentery, constipation* | 25●,28●,11♦,21♦,23♦,24♦,26♦ | Boiled leaves and fruits are eaten for the treatment of dysentery and constipation. | Cm | 2 | 0.09 | 22 | 0.13 | 0.39 |
| *Centella asiatica* (L.) Urb. ; MCT-F-075  Least Concern | Apiaceae | Thankuni | Nangdaissa/M | N | H | WP | W | *Fever,* *loss of smell and taste , carbuncles, dysentery* | 20●,21●,24●,26●,27●,28●,30●, 19♦,18♦,23♦ | A paste of the whole plant is applied to the head during fever and also applied to the affected area in carbuncles. Juice of the whole plant is taken at a dose of one teaspoonful twice daily for three days in fever and dysentery. The whole plant, smashed with tamarind, is taken to correct loss of smell and taste*.* | Cm | 4 | 0.15 | 26 | 0.15 | 0.58 |
| *Cheilocostus speciosus* (J. Koenig) C. D. Specht; MCT-F-011 | Costaceae | Keu | Praing-demu/C | E | S | L | W | *Asthma* | 23●,22♦ | An infusion of the leaves is taken at a dose of about one cupful twice daily for seven days. | O | 1 | 0.05 | 22 | 0.13 | 0.33 |
| *Chenopodium album* L. ; MCT-F-097 | Amaranthaceae | Betua shak | Bra-tho-aa/M | N | H | WP | W | *Indigestion* |  | The whole plant is cooked along with ginger (*Zingiber officinale*), and taken every morning for five to ten days. | Cm | 1 | 0.20 | 5 | 0.03 | 0.13 |
| *Chromolaena odorata* (L.) R.M. King & H. Rob.; MCT-F-039 | Asteraceae | Asamlata | Welemra/C; ohipanea/M; desmara kher/T | N | H | L | W | *Cuts and wounds, snake bites* |  | A paste of the leaves is applied directly to cuts and wounds by /M, /C and /T indigenous communities. Juice of the leaves is applied to the affected area in snake bites (Green color) by /M communities. | A | 1 | 0.05 | 20 | 0.11 | 0.30 |
| *Cleome viscosa*  L.; MCT-F-089 | Cleomaceae | Atha hurhuria | Unknown/T | N | H | L | W | *High Blood pressure* | 4● | A maceration of leaves in boiling water is taken to reduce high blood pressure. | O | 1 | 0.12 | 8 | 0.04 | 0.17 |
| *Clerodendrum* *indicum* (L.) Kuntze; MCT-F-129 | Lamiaceae | Banchat | Nerumby/M; ; bainpara gach/T | N | S | R | W | *To remove poisonous substances from the body, Diarrhea, knee pain, jaundice* | 27●,22♦,25♦,26♦ | The root is rubbed on stone to release sap, which is taken to remove poisonous substances from the body in cases of deliberate self-poisoning (/M). Juice of the root is taken with sugar three times daily until cure of diarrhea and jaundice (/M). Baked roots are tied around the knee to reduce the knee pain (/T). | A | 4 | 0.21 | 19 | 0.11 | 0.50 |
| *Clerodendrum infortunatum* L; MCT-F-020 | Lamiaceae | Bhat | Khaungkhaba/M | N | S | L, Fw | W | *Boils, gastritis, stomachache, diarrhea* | 23♦ | Young powdered leaves are boiled with water and the extract is taken twice daily for one month to cure gastritis. Young leaves are eaten with green chili to treat gastritis (breathing should be suppressed while taking this treatment). Boiled leaves are added to bath water in the treatment of boils. Young leaves are taken to reduce stomachache. A paste of the flowers along with black cumin (*Nigella sativa*) is taken at a dose of half a cupful daily for three days in diarrhea. | A | 4 | 0.18 | 22 | 0.13 | 0.54 |
| *Clitoria ternatea* L.; MCT-F-127 | Fabaceae | Aparajita | Ongmaiyo/T;amio/M | N | C | Fw, R, Sd | C | *Pneumonia, cough,*  *Lactation, Measles, diuretic* | 23●,21♦,30♦ | Flowers are baked on leaves of *Musa sapientum* during cooking of rice, and the resultant juice is taken at a dose of one teaspoonful twice daily for seven days to cure pneumonia and cough (/T). A paste of the root is taken (as much as possible) to enhance the process of lactation and as a diuretic. The white-colored seeds are given to children as a preventive measure for measles (/M). | O | 5 | 0.21 | 24 | 0.14 | 0.64 |
| *Codariocalyx* *motorius* (Houtt.) H.Ohashi; MCT-F-143 | Fabaceae | Gorachand | Ang-gera/M | E | H | R | W | *Stones in the urinary bladder* |  | A decoction of the root is taken at a dose of one teaspoonful twice daily for three days to remove stones from the urinary bladder. | Rr | 1 | 0.07 | 15 | 0.09 | 0.25 |
| *Congea tomentosa* Roxb.; MCT-F-152 | *Lamiaceae* | Kongia | Lummu, Crimui/M; not known/C/T | N | T | S, Fw, L | W | *Tumor, allergy, cancer* |  | Sap of the stem is applied onto external tumors, and left for at least seven days (/C). The juice of both leaves and flowers is taken at a dose of two teaspoonfuls three times daily for one month to treat cancer (/M and /T). A paste of the leaves is applied to the interdigital region to treat allergic problems. | Rr | 2 | 0.40 | 5 | 0.03 | 0.20 |
| *Coriandrum sativum* L.; MCT-F-098 | Apiaceae | Dhaniya | Unknown/T | N | H | WP | C | *Inflammation* | 17●, 10♦,28♦ | The whole plant is ground with mustard oil and smeared on affected areas of inflammation. | Cm | 1 | 0.05 | 21 | 0.12 | 0.32 |
| *Crotalaria juncea* L.; MCT-F-100 | Fabaceae | Shonpat | Pao-oyai-pang/M | N | H | R | W | *Insanity* | 26♦ | The root extract is taken along with other ingredients (unknown) to treat insanity. | Cm | 1 | 0.06 | 16 | 0.09 | 0.26 |
| *Curculigo orchioides* Gaertn; MCT-F-135 | Hypoxidaceae | Talamuli | Unknown/T | N | H | Bb | W | *Skin diseases, leucorrhoea* | 25●, 22♦ | A paste of the bulb with cow’s milk is taken at a dose of two teaspoonfuls three times daily during menstruation for leucorrhoea. The same formulation is smeared around the vaginal area, preferably early in the morning and at night, to treat leucorrhoea. A paste of the leaves and honey are applied to affected areas in skin diseases. | Rr | 2 | 0.25 | 8 | 0.04 | 0.24 |
| *Curcuma caesia* Roxb.; MCT-F-079 | Zingiberaceae | Kalo hulud | Nangwar-kha/M | N | H | Rz | W | *Gastritis* |  | The rhizome is eaten to cure gastritis. | O | 1 | 0.14 | 7 | 0.04 | 0.15 |
| *Curcuma longa* L.; MCT-F-132 | Zingiberaceae | Hulud | Kakung/C; chith nu/M; Olud/T | N | H | WP, Rz, L | C | *Scabies, Malaria, chicken pox, blood purifier* | 12●,15●,21●,24●,26●,30●,17♦,28♦ | The whole plants are crushed together with fruits of *Nigella sativa* and honey, and smeared on affected areas to treat scabies (/C). The juice of the rhizome is taken at a dose of one teaspoonful twice daily for fifteen days to treat malaria and chicken pox (/M). Young leaves are eaten as a blood purifier (/T). | Cm | 4 | 0.15 | 26 | 0.15 | 0.58 |
| *Curcuma zedoaria* (Christm.) Roscoe; MCT-F-128 | Zingiberaceae | Soti | Kaikazikai/C | N | H | Rz | W | *To remove poison from the stomach* |  | Sap of the rhizome is eaten in cases of ingestion of poison. | O | 1 | 0.05 | 21 | 0.12 | 0.32 |
| *Cucumis sativus* L.; MCT-F-048 | Cucurbitaceae | Khira | Mou-prishi-aa-rwoM | N | C | Fr | C | *Diarrhea* | 5●,26♦ | If fruits are taken daily (as much as possible), they are believed to cure diarrhea. | Cm | 1 | 0.08 | 12 | 0.07 | 0.21 |
| *Cuscuta reflexa* Roxb.; MCT-F-092 | Cuscutaceae | Swarnalata | Jigro/M | N | C | WP | W | *To increase longevity* | 20♦,24♦,28♦ | Juice of the whole plant, along with honey and two other unknown ingredients, is taken to increase longevity. | Rr | 1 | 0.05 | 18 | 0.10 | 0.28 |
| *Cyanthillium cinereum*  (L.) H. Rob.; MCT-F-154 | Asteraceae | Shial lata | Hung fu/M | N | H | R, WP | W | *Tumors, Rheumatism, Boils, piles* | 25● | A decoction of the root is taken at a dose of one teaspoonful three times daily for ten-fifteen days to treat tumors, rheumatism and boils. A paste of the whole plant is applied to piles. | O | 4 | 0.18 | 22 | 0.13 | 0.54 |
| *Cymbidium aloifolium* (L.) Sw.; MCT-F-060 | Orchidaceae | Tosabak | Tethalangni/M | N | H | L | W | *Ear infections, Boils in ear* |  | Sap from warmed leaves is applied at a dose of two-three drops into the ear, to cure ear infections or boils. | O | 2 | 0.14 | 14 | 0.08 | 0.30 |
| *Cymbopogon flexuosus* (Nees ex Streud.) W. Watson; MCT-F-040 | Poaceae | Ghadhatrina | Chabalan apan/M | N | H | R | C | *Cuts and wounds* | 20♦,27♦ | Root sap is applied directly to cuts and wounds. | Rr | 1 | 0.25 | 4 | 0.02 | 0.12 |
| *Cyperus rotandus* L.; MCT-F-159 | Cyperaceae | Mutha | Boutu-mra/M | N | H | R | W | *Wounds on lips* | 26♦,28♦ | The root is taken to cure wounds on the lips and mouth. | Cm | 1 | 0.08 | 12 | 0.07 | 0.21 |
| *Dalbergia oliveri* Prain; MCT-F-001  Endangered | Fabaceae | Unknown | Chimongsoi/M | N | T | Fw,R,L | W | *Abdominal pain* |  | Juice from flowers, roots and leaves is taken at a dose of a cupful three times daily for ten days to cure abdominal pain. | O | 1 | 0.04 | 23 | 0.13 | 0.35 |
| *Dalbergia stipulacea* Roxb.; MCT-F-064 | Fabaceae | Dadbari | Chimonggoi, challi/M | N | T | S,L,Fr,R | W | *Eczema; Diarrhea, general weakness, piles* |  | Powdered stems, leaves and fruit are rubbed on the affected areas in eczema. Powdered stems, leaves and fruits are taken with water *ad libitum* to treat diarrhea and general weakness. A paste from the leaves and root is taken twice daily for seven days to cure piles. | O | 4 | 0.17 | 24 | 0.14 | 0.57 |
| *Datura metel* L.; MCT-F-101 | Solanaceae | Dhutara | Dhutura gaith/T | N | S | L | W | *Insect bites* | 23●,22♦,25♦,28♦,30♦ | Crushed leaves are smeared on the affected areas. | O | 1 | 0.06 | 16 | 0.09 | 0.25 |
| *Dendrophthoe falcata*  (L.f.) Ettingsh.; MCT-F-030 | Loranthaceae | Pharulla | Chupru, Kheyagainadu/M | N | S | S,L, R | W | *Cancer, tumor, fevers* | 23♦,25♦ | An extract of leaves and stems is taken to cure cancer and tumors. Sap of the root is rubbed on the whole body if suffering from fever. | Rr | 3 | 0.12 | 24 | 0.14 | 0.49 |
| *Desmodium heterocarpon* (L.) DC.; MCT-F-155 | Fabaceae | Karpo modi | Somorekhuyatu/M | N | H | R | W | *Tumor, urinary disorders* | 18●, 20♦ | The root is rubbed on a stone and the resultant sap is taken twice daily for one month to treat stomach tumors and urinary disorders. | O | 2 | 0.12 | 16 | 0.09 | 0.33 |
| *Drimia indica*  (Roxb.) Jessop; MCT-F-002 | Asparagaceae | Bonpeaj | Tokrapiai/M | N | H | Rz | W | *Abdominal pain in children* | 26♦ | The rhizome is tied around the waist to reduce abdominal pain in children. | O | 1 | 0.09 | 11 | 0.06 | 0.20 |
| *Duabanga grandiflora* (DC.) Walp.; MCT-F-029 | Lythraceae | Bandorhola | Miyonggoufa/M; not known/C/T | E | T | L,S,B | W | *Cancer, boils, tumors* |  | An extract of leaves and young stems combined with unknown ingredients is taken at a dose of one teaspoonful three times daily for two-three months to treat cancer (/M and /T). A decoction prepared from leaves, along with the root sap of *Achyranthes aspera* and honey is given to treat cancer by /C communities. A paste of the bark is used to cure boils. A decoction of leaves is taken to treat tumors (/M). | Rr | 3 | 0.43 | 7 | 0.04 | 0.29 |
| *Eclipta prostrata* (L.) L.; MCT-F-013  Least Concern | Asteraceae | Kalokeshi | Mingkheya-aa/M | N | H | WP | W | *Beautification of hair and teeth, longevity, albinism* | 26♦,27♦ | A paste of the whole plant is applied to the hair to increase its beauty as a hair colorant. The powdered whole plant, along with seeds of *Nigella sativa* and cow’s milk, are taken at a dose of one cupful daily for three months to dye hair black. The powdered whole plant is taken with cow’s milk to increase beauty of the teeth. The powdered whole plant, along with seeds of *Nigella sativa,* the fruit of *Averrhoea bilimbi* and other ingredients, is taken at a dose of one teaspoonful daily to increase longevity. The powdered whole plant, the root of *Terminalia chebula* and the fruit sap of *Citrus aurantiifolia* are applied to affected areas in albinism. | O | 3 | 0.11 | 26 | 0.15 | 0.51 |
| *Engelhardtia spicata* Lechen.*ex* Blume; MCT-F-153  *Lower Risk/Least Concern* | Juglandaceae | Jumkavadi | Minggeuri/C ; Chungpaila/M; not known/T | N | T | S, L, | W | *Tumors, Breast cancer* |  | A paste of the leaves and stems is rubbed on external tumors until cure by the /M and /C communities. If a tumor is seen inside the breast, then a paste of leaves and stems is taken along with other unknown ingredients. (/T). | Rr | 2 | 0.28 | 7 | 0.04 | 0.22 |
| *Enydra fluctuans* DC.; MCT-F-145  Least Concern | Asteraceae | Helencha | Unknown /C | N | H | WP | W | *Swellings* | 28●,27♦ | A paste of the whole plant is applied to swellings. | Cm | 1 | 0.17 | 6 | 0.03 | 0.14 |
| *Equisetum* *ramosissimum* Desf.; MCT-F-084 | Equisetaceae | Bash ghash | Unknown/T; /C | N | H | Rz | W | *Gastritis, abdominal pain and paralysis* | 6♦ | An extract of the rhizome is taken in gastritis (/T). The baked rhizome is massaged on the lower abdomen to obtain relief from abdominal pain and also used in cases of paralysis (/C). | Rr | 3 | 0.21 | 14 | 0.08 | 0.37 |
| [*Erigeron* *floribundus* (Kunth) Sch.Bip.](http://www.theplantlist.org/tpl1.1/record/gcc-120553); MCT-F-122 | Asteraceae | florigeron | Unknown/T | E | H | R | W | *Piles* |  | The powdered root is taken along with rice-washed water at a dose of one cupful daily for three days. | Rr | 1 | 0.11 | 9 | 0.05 | 0.25 |
| *Eryngium foetidum* L.; MCT-F-131 | Apiaceae | Bilati dhone | Unknown /C | N | H | WP | W | *Ringworm* |  | A paste from the whole plant is applied to the infected area. | O | 1 | 0.12 | 8 | 0.04 | 0.17 |
| *Erythrina suberosa* Roxb.; MCT-F-158 | Fabaceae | Madar | Unknown /C | N | T | L | W | *Urticaria* |  | A paste prepared from the leaves is applied directly to the affected area. | O | 1 | 0.10 | 10 | 0.06 | 0.19 |
| *Eucalyptus globulus*  Labill; MCT-F-008 | Myrtaceae | Eucalyptus | Unknown/M | E | T | L | C | *Analgesic, coughs* | 1●,9●,24♦,28♦ | A decoction of leaves, if taken at a dose of one teaspoonful daily for five days, is believed to act as analgesic. Smoking of the leaves is believed to treat cough. | Cm | 2 | 0.13 | 15 | 0.09 | 0.32 |
| *Ficus auriculata* Lour.; MCT-F-053 | Moraceae | Barodumur | Unknown /C | N | T | Fr | W | *Dysentery* | 18● | Ripe fruits are eaten, while unripe fruits are cooked with small fish and taken to treat dysentery. | Rr | 1 | 0.05 | 21 | 0.12 | 0.32 |
| *Ficus hispida* L.f.; MCT-F-049 | Moraceae | dumur | Pa-sha-ting/M | N | T | Fr | W | *Diarrhea* |  | Three to four fruits are taken for the treatment of diarrhea. | O | 1 | 0.08 | 12 | 0.07 | 0.21 |
| *Ficus racemosa* L. var. *racemosa;*  MCT-F-055 | Moraceae | Jug dumur | Jabuna/M | N | T | Fr | W | *Dysentery, diabetes, carminative* | 25● | Ripe fruits are eaten, while unripe fruits are cooked and taken (as much as possible) to treat dysentery and diabetes. Juice of the fruit is taken as a carminative. | O | 3 | 0.13 | 23 | 0.13 | 0.48 |
| *Ficus semicordata*  Buch.-Ham.*ex* Sm.; MCT-F-107 | Moraceae | Sadimadi dumur | Baaung fung/C | N | T | Fr | W | *Jaundice* |  | The fruit juice, along with ginger (*Zingiber officinale*) and the fruit of *Tamarindus indica*, is given at a dose of one cupful three times daily for ten to fifteen days in cases of jaundice. | Rr | 1 | 0.08 | 13 | 0.07 | 0.22 |
| *Grewia villosa* Wild.; MCT-F-016 | Malvaceae | Banta bicha | Unknown/C | E | S | Fr | W | *Boils* | 16● | The fruit paste is applied to boils. | Rr | 1 | 0.20 | 5 | 0.03 | 0.13 |
| *Hedychium villosum* Wall.; MCT-F-099 | Zingiberaceae | Pashmi ada | Unknown/T | N | H | Rz | W | *Injuries* | 19● | The rhizome juice is externally applied to injuries. | Rr | 1 | 0.20 | 5 | 0.03 | 0.13 |
| *Hedyotis scandens* Roxb.; MCT-F-037 | Rubiaceae | Latakani | Unknown/M | N | H | L | W | *Coughs, Inflammation* | 19● | Leaf juice is taken at a dose of two-three drops twice daily for three days to treat cough in children. A paste from the leaves is applied to affected areas to treat inflammation. | O | 2 | 0.22 | 9 | 0.05 | 0.25 |
| *Helminthostachys zeylanica* (L.) Hook.; MCT-F-080 | Ophioglossaceae | Krimi fern | Unknown/T | E | H | WP | W | *Gastritis* |  | Dry powdered whole plant is taken with honey to treat gastritis. | Rr | 1 | 0.25 | 4 | 0.02 | 0.12 |
| *Hemidesmus indicus*  [(L.) R. Br. ex Schult.](http://www.theplantlist.org/tpl1.1/record/tro-2609794); MCT-F-112 | Apocynaceae | Anontomul | Unknown/C/T | N | H | R | W | *Leucorrhoea, Malaria, rheumatism* | 15●,23●,25●,30♦ | The root paste is applied around the vaginal area during menstruation for the treatment of leucorrhoea (/C). The root juice, along with the juice of *Azadirachta indica* leaves, is taken *ad libitum* to cure malaria. The same preparation is taken at a dose of one teaspoonful twice daily for rheumatism (/T). | Rr | 3 | 0.25 | 12 | 0.07 | 0.35 |
| *Hibiscus rosa-sinensis* L.; MCT-F-104 | Malvaceae | Joba | Soiha/M | N | S | R, Fw | C | *Irregular menstruation, constipation, boils* | 28●,21♦,22♦,24♦ | Juice of the root is taken to treat irregular menstruation and constipation. A paste of the flowers is smeared on boils. | Cm | 3 | 0.12 | 24 | 0.14 | 0.49 |
| *Hibiscus sabdariffa* L. var. *sabdariffa* ; MCT-F-094 | Malvaceae | Lalmesta | Koropata/M | N | H | Fr, L | W | *To increase sexual desire, Diabetes, coughs & colds* | 14●,11♦,17♦,22♦ | The fruit is taken along with honey to increase sexual desire. An extract of boiled leaves is taken in diabetes and to treat coughs and colds. | O | 3 | 0.17 | 18 | 0.10 | 0.42 |
| *Hippochaete* *debilis* (Roxb. ex Vaucher) Ching; MCT-F-026 | Equisetacea | Unknown | Chakrin niai/C; peulacha/M | E | H | WP | W | *Boils; fungal infections; abdominal pain, baldness* | 19♦ | A paste from the whole plant is applied to boils and fungally infected areas; a paste with rice-washed water is taken at a dose of one cupful daily for ten days to treat abdominal pain (/C). A powder prepared from the whole plant, along with chicken fat, is massaged to bald regions of scalp to promote hair growth (/M). | Rr | 4 | 0.21 | 19 | 0.11 | 0.50 |
| *Holarrhena pubescens* Wall. ex G. Don; MCT-F-088 | Apocynaceae | Kurchi | kuruk/T | N | S | B, Fw | W | *Headache, snake bite* | 23●,25●,27♦ | A paste from the bark is rubbed on the forehead to afford relief from headache. Juice of the flowers is given in cases of snake bite. | O | 2 | 0.09 | 21 | 0.12 | 0.39 |
| *Hypserpa nitida* Miers ex Benth.; MCT-F-147 | Menispermaceae | Serpa | Debrachi/M | N | S | R,L | W | *Toothache, haemostatic* |  | Root sap is used for toothache and a paste of root and leaves is applied to stop bleeding. | O | 2 | 0.08 | 24 | 0.14 | 0.42 |
| *Hyptis brevipes* Poit.; MCT-F-045 | Lamiaceae | Gol tukma | Unknown /C | N | H | Sd | W | *Diabetes* |  | About 5-10 gram of seed, if taken daily on an empty stomach especially early in the morning, is believed to cure diabetes. | O | 1 | 0.04 | 22 | 0.13 | 0.33 |
| *Hyptis suaveolens* (L.) Poit.; MCT-F-156 | Lamiaceae | Tukma | Unknown /C | N | H | L, Fr | W | *Ulcers, Eczema* | 12●,30●,27♦ | The fruit juice, along with the fruit of *Citrus aurantifolia*, is taken to treat ulcers. A paste of the leaves is applied to the affected areas in eczema. | Cm | 2 | 0.07 | 27 | 0.15 | 0.46 |
| *Ichnocarpus frutescens* [(L.) W.T.Aiton](http://www.theplantlist.org/tpl1.1/record/kew-102359); MCT-F-034 | Apocynaceae | Samalata | Bhutta ludi/T | N | H | L | W | *Constipation* | 22♦ | The leaves are made into a decoction and given at a dose of one teaspoonful twice daily to treat constipation, especially for children aged 10-15 years. | Rr | 1 | 0.08 | 13 | 0.07 | 0.22 |
| *Ixora nigricans* R. Br. *Ex* Wight & Arn.; MCT-F-009 | Rubiaceae | Kuti rongon | Unknown /C | N | H | R | W | *Anthelmintic* | 27♦ | The root is chewed along with garlic as an anthelmintic. | Cm | 1 | 0.07 | 14 | 0.08 | 0.23 |
| *Jacquemontia paniculata* (Burm.f.) Hallier f.; MCT-F-133 | Convolvulaceae | Montilata | Unknown /C | N | C | WP | W | *Skin disease* |  | Bathing with the whole plant decoction is believed to cure skin disease. | Cm | 1 | 0.05 | 18 | 0.10 | 0.28 |
| *Jatropha* *gossypiifolia* var. *elegans* (Pohl) Müll.Arg.; MCT-F-123 | Euphorbiaceae | Lal bherenda | Karachuni/M | E | S | L | W | *Piles* |  | A paste of the young leaves is applied to the anus to treat piles. | O | 1 | 0.09 | 11 | 0.06 | 0.20 |
| *Justicia adhatoda* L.; MCT-F-012 | Acanthaceae | Basak | Paying-heu/M | N | S | L | C | *Asthma, coughs* | 19●,22●,23●,24●,26●,27●,28●,30●,15♦,21♦ | Juice of young leaves is taken at a dose of one cupful daily to treat cough and asthma. | Rr | 2 | 0.07 | 30 | 0.17 | 0.49 |
| *Justicia gendarussa* Burm.f.; MCT-F-086 | Acanthaceae | Jagatmadan | Chonhaching guite/T | N | S | L | W | *Gout* |  | A paste of the leaves is applied in gout. | Cm | 1 | 0.08 | 13 | 0.07 | 0.22 |
| *Lactuca sativa*  L.; MCT-F-010 | Asteraceae | Kahu | Unknown/T | E | H | L | C | *Antiseptic* | 6● | Young leaves are directly rubbed on any injuries as an antiseptic | Cm | 1 | 0.10 | 10 | 0.06 | 0.19 |
| *Lawsonia inermis* L.; MCT-F-019 | Lythraceae | Mehedi | Unknown /C | N | T | L | C | *Boils, beautification of hair and nail.* | 21●,22●,30♦ | A paste of the leaves is smeared on boils. The same preparation is applied to nails and hair for beautification purposes. | O | 2 | 0.06 | 35 | 0.20 | 0.55 |
| *Leucas aspera* (Roth) Spreng; MCT-F-052 | Lamiaceae | Shetodron | Payingdungcha, sagrain/M | N | H | WP | W | *Dog bites; abdominal pain, gastritis, piles, asthma, fever* | 25●,27●,23♦,26♦ | Juice of the whole plant is taken at a dose of one teaspoonful three times daily for five days for abdominal pain; the same dose is taken on an empty stomach for ten days for the treatment of gastritis; an extract of the whole plant is taken at a dose of two teaspoonfuls three times daily for one month alongside other unknown ingredients to treat dog bites. A paste of the plant is also rubbed on affected areas in cases of dog bite. A paste of the whole plant is rubbed on the head of children if they suffer from asthma and fever. A paste of the whole plant is also taken at a dose of one spoonful twice daily to cure piles. | Cm | 6 | 0.17 | 35 | 0.20 | 0.83 |
| *Lepisanthes senegalensis* (Poir.) Leenh.; MCT-F-149 | Sapindaceae | Banlichu | Unknown /C | N | S | L | W | *Tumors* |  | A decoction prepared from the leaves is taken along with goat’s milk to treat tumors. | Rr | 1 | 0.05 | 21 | 0.12 | 0.32 |
| *Leucas zeylanica* [(L.) W.T.Aiton](http://www.theplantlist.org/tpl1.1/record/kew-111936); MCT-F-067 | Lamiaceae | Dondokalosh | Paithuncha/M | N | H | WP | W | *Excessive bleeding during childbirth* |  | Juice of the whole plant is taken at a dose of one teaspoonful twice daily to reduce hemorrhages. | Rr | 1 | 0.04 | 25 | 0.14 | 0.36 |
| *Litsea glutinosa*  (Lour.) C.B. Rob.; MCT-F-028 | Lauraceae | Mentha pata | Udunaispeye/C | E | S | B | W | *Cancer* |  | Powdered bark is applied to the affected areas in cancer. | Rr | 1 | 0.06 | 16 | 0.09 | 0.26 |
| *Lygodium flexuosum* (L.) Sw. ; MCT-F-021 | Lygodiaceae | Saralata fern | Makala/M | N | H | L | W | *Boils, to increase sexual desire* |  | A paste of the leaves is applied to boils. A decoction of leaves, along with honey, is taken to increase sexual desire. | O | 2 | 0.07 | 28 | 0.16 | 0.47 |
| *Maesa indica* (Roxb.) A.DC.; MCT-F-134 | Primulaceae | Ramjoni | Tolcheri/M | N | H | Fw, L,S | W | *Skin diseases* |  | A paste of the flowers, leaves and stems is rubbed on the skin to cure skin diseases. | O | 1 | 0.07 | 14 | 0.08 | 0.23 |
| *Maesa ramentacea* (Roxb.) A.DC.; MCT-F-111 | Primulaceae | Moricha | Sebbigri/M | N | S | B | W | *Leucorrhoea* |  | A paste of the bark is taken, with other unknown ingredients, for the treatment of leucorrhoea. | O | 1 | 0.06 | 17 | 0.10 | 0.27 |
| *Matricaria chamomilla* L.; MCT-F-033 | Asteraceae | Chamomile | Unknown/T | E | H | L, Fw | C | *Colic, sedative* | 6● | Juice of the leaves is used as a sedative. An infusion of flowers is given to children to treat colic. | O | 2 | 0.33 | 6 | 0.03 | 0.21 |
| *Merremia vitifolia* (Burm.f.) Hallier f.; MCT-F-017 | Convolvulaceae | Kormolata | Talingruf/C | N | H | L | W | *Boils* |  | A paste of leaves is applied to boils. | Cm | 1 | 0.06 | 17 | 0.10 | 0.27 |
| *Micromelum minutum* (J.G. forster) Wight & arn.; MCT-F-076 | Rutaceae | Ghaskhasa | Unknown /C | N | S | L | C | *Fever; dehydration, chest pain* |  | A decoction of leaves, mixed with a decoction of *Phaseolus vulgaris,* is taken at a dose of about 3 teaspoons three times daily for five to seven days in cases of fever and dehydration. A paste of the leaves is rubbed on the chest to relieve chest pain. | Rr | 3 | 0.14 | 22 | 0.13 | 0.47 |
| *Mimosa pudica* L.; MCT-F-024  Least Concern | Mimosaceae | Lozzaboti | Rapaing/M | N | H | WP | W | *Boils, toothache, as a source of multivitamins, and ulcers* | 26●,27●,28●,30●,23♦ | A paste of the whole plant is rubbed onto boils, after rubbing away all waste materials exuding from the lesion. A paste of the whole plant is also used to cure toothache. Juice from the whole plant is taken as a source of multivitamins. This same preparation, along with other unknown ingredients, is given *ad libitum* to treat ulcers. | Cm | 3 | 0.08 | 39 | 0.22 | 0.67 |
| *Mitragyna parvifolia* (Roxb.) korth; MCT-F-151 | Rubiaceae | Kelikadam | Takhaba, pangkhabong/M | N | T | R, B,L | W | *Tumors* | 25♦ | The root sap is taken at a dose of one teaspoonful three times daily for one month to treat tumors. A paste of the bark and leaves is also used to treat tumors. | O | 1 | 0.07 | 15 | 0.09 | 0.25 |
| *Morinda umbellata* L.; MCT-F-138 | Rubiaceae | Gassa latha | Toriru/M | N | H | L, Fw | W | *Stomachache* |  | A decoction of the boiled leaves and flowers is taken at a dose of two teaspoonfuls twice daily for five days to treat stomachache. | Cm | 1 | 0.07 | 14 | 0.08 | 0.24 |
| *Mucuna pruriens* (L.) DC. ; MCT-F-114 | Fabaceae | Bichchoti | Ole/M | N | C | R, Sd | W | *Malaria, impotence, uterine disorders, infertility* | 23● | The boiled root is taken daily for three days in malaria. The root is boiled with water (water:root-3:1) and taken as a half cup twice daily for fifteen days in infertility and uterine disorders. The powdered seed, along with honey and cow’s milk, is taken at a dose of one teaspoonful three times daily for one month to treat impotence. | O | 4 | 0.19 | 21 | 0.12 | 0.53 |
| *Musa paradisiaca* L.; MCT-F-157 | Musaceae | Aittakola | Nwpupi/M | N | H | R | C | *Urinary problems (Dysuria)* | 26♦ | Juice of the root, along with sugar, is taken at a dose of one teaspoonful twice daily for seven days to treat dysuria. | Cm | 1 | 0.09 | 11 | 0.06 | 0.20 |
| *Mussaenda glabra* Vahl; MCT-F-068 | Rubiaceae | Bichmali | Chupru/M | N | S | R | W | *Excessive bleeding during childbirth* |  | The root sap is taken at a dose of one teaspoonful twice daily. | O | 1 | 0.05 | 21 | 0.12 | 0.32 |
| *Mycetia longifolia* (Wall.) Kuntze; MCT-F-150 | Rubiaceae | Mycetelon | Unknown/T | N | S | R | W | *Tumors* |  | A decoction of the root is taken as one cupful daily for fifteen days to treat internal tumors. | Rr | 1 | 0.07 | 14 | 0.08 | 0.23 |
| *Nanorrhinum* *ramosissimum* (Wall.) Betsche; MCT-F-108 | Plantaginaceae | Unknown | Unknown/C | E | H | L | W | *Jaundice* |  | The leaves are cooked with crabs and the prepared soup is taken at a dose of one cupful three times daily for ten days. | Rr | 1 | 0.20 | 5 | 0.03 | 0.13 |
| *Nicotiana tabacum* L.; MCT-F-124 | Solanaceae | Tamak | Staa-tak/C | E | H | L | C | *Piles* | 2♦ | A paste of the leaves is smeared on the anus to treat piles. | A | 1 | 0.06 | 17 | 0.10 | 0.27 |
| *Nigella sativa*  L.; MCT-F-031 | Ranunculaceae | Kalojira | Unknown/M | E | H | Sd | C | *Cardiovascular disease, diabetes* | 17●, 24♦,26♦,28♦ | About 5-8 gram of seeds are eaten once daily, preferably early in the morning on an empty stomach, for cardiovascular disease and diabetes. | Rr | 2 | 0.10 | 20 | 0.11 | 0.37 |
| *Ocimum tenuiflorum* L. MCT-F-072 | Lamiaceae | Kalatulsi | Kalatulsi/T | N | H | L | W | *Fever, cough* | 28● | An extract of the leaves is taken at a dose of one cupful twice daily for seven days to cure fever and cough. | O | 2 | 0.09 | 23 | 0.13 | 0.41 |
| *Oxalis corniculata* L.; MCT-F-063 | Oxalidaceae | Amrul | Mring blu/M | N | H | L, WP | W | *Eczema, piles* | 21●,30●,10♦,28♦ | A paste of the leaves is applied to the affected areas in eczema. A decoction of the whole plant is taken to treat piles. | O | 2 | 0.09 | 21 | 0.12 | 0.39 |
| *Passiflora edulis* Sims; MCT-F-018 | Passifloraceae | Tang jhumka | Baktainche/C | E | H | L | W | *Boils* | 13♦,17♦ | Powdered leaves are applied to the affected areas in boils. | Cm | 1 | 0.08 | 12 | 0.07 | 0.21 |
| *Passiflora foetida* L.; MCT-F-106 | Passifloraceae | Jumkolata | Powmachi/M; mathri gula/T | E | C | L | W | *Itch, bronchitis* | 12●,30●,22♦ | Leaves are directly rubbed onto the affected areas in itch (/M). Juice of the leaves is taken to treat bronchitis (/T). | A | 2 | 0.06 | 32 | 0.18 | 0.51 |
| *Piper retrofractum* Vahl; MCT-F-007 | Piperaceae | Choitro | Vutpan/T | N | C | L | C | *Allergy* |  | Leaves are boiled, and the resulting leaf water is used for bathing in cases of allergy. | Rr | 1 | 0.11 | 9 | 0.05 | 0.18 |
| *Plumbago indica* L.; MCT-F-118 | Plumbaginaceae | Raktachita | Unknown /C | N | H | S | W | *Migraine* | 22♦ | Several drops of the stem decoction are poured into the nasal cavity to treat migraine. | O | 1 | 0.05 | 20 | 0.11 | 0.30 |
| *Plumeria rubra* L.; MCT-F-023 | Apocynaceae | Golokchapa | Angara/M; chechena/T; aangropao/C | E | T | L, B | W | *Boils, Toothache* |  | Leaf sap is used to treat boils in both /M and /T communities. Juice of the bark is taken for toothache (/C). | Rr | 2 | 0.17 | 12 | 0.07 | 0.28 |
| *Pogostemon benghalensis* (Burm.f.) Kuntze; MCT-F-119 | Lamiaceae | Juilata | Crongbei ing/M | N | H | WP | W | *Paralysis* |  | The whole plant is kept in a piece of cloth, warmed and applied to affected areas to treat paralysis. | O | 1 | 0.08 | 12 | 0.07 | 0.21 |
| *Psidium guajava* L.; MCT-F-050 | Myrtaceae | Peyara | Gayoungci/C | N | T | L | C | *Diarrhea,* | 12●,13●,19●,28●,24♦ | Juice of the leaves is taken to treat diarrhea. | Cm | 1 | 0.05 | 21 | 0.12 | 0.32 |
| *Pueraria tuberosa* (Willd.) DC.; MCT-F-090 | Fabaceae | Gola kunch | Unknown /C | E | C | Tb | W | *High blood pressure* |  | A paste prepared from the tubers is taken at a dose of half a teaspoon three times daily for seven days to control high blood pressure. | Rr | 1 | 0.08 | 12 | 0.07 | 0.21 |
| *Rauvolfia serpentina* (L.) Benth. *Ex* Kurz; MCT-F-091 | Apocynaceae | Swarpagondha | Bummaraza/T | N | H | R,S | W | *High blood pressure, snake bite, stomachache* | 22●,28●,30●,15♦,27♦ | An extract of the root is taken at a dose of one teaspoonful twice daily for fifteen days to control high blood pressure, while the same dose is taken in snake bite. A paste of the stem with honey is taken for the treatment of stomachache. | Rr | 3 | 0.07 | 43 | 0.25 | 0.71 |
| *Ricinus communis* L.; MCT-F-125 | Euphorbiaceae | Verenda | Enonggai/T | N | T | L, Fr | W | *Piles, fever, cough, carbuncles, ulcers, Stomachache, constipation* | 9●,10●,12●,13●,21●,27●,30●,6♦,8♦, 11♦,14♦,16♦,17♦,19♦,26♦ | Warm leaves are briefly placed on the anus to treat piles. Juice of the leaves is taken at a dose of one cupful twice daily until cure of fever and cough, and the same form is taken twice daily for 15 days before food in the treatment of ulcers. Oil extracted from flowers is applied to carbuncles. Juice of the leaves, along with coconut oil, is taken to treat stomachache and constipation. | O | 7 | 0.32 | 22 | 0.13 | 0.76 |
| *Sansevieria trifasciata* Prain; MCT-F-061 | Asparagaceae | Sapahara | Unknown /C; /T | E | H | L | W | *Earache, blood purifier* |  | One to two drops of warm leaf sap is poured into the ear to treat earache (/C). A decoction prepared from young leaves is taken once daily as a blood purifier (/T). | Rr | 2 | 0.08 | 26 | 0.15 | 0.44 |
| *Scoparia dulcis* L.; MCT-F-057 | Plantaginaceae | Bandhuni | Granza/granzaba/M | N | H | WP | W | *Dysentery, vomiting with blood, Urinary problems, toothache, abdominal pain, insect bites* | 12●,27●,3♦,25♦ | The powdered whole plant is taken with sugar twice daily for seven days for the treatment of dysentery and vomiting. The juice of the whole plant, along with rice-washed-water and sugar, is taken at a dose of one teaspoonful twice daily to treat irregular excretion, toothache and abdominal pain. A paste prepared from the whole plant is applied to the affected area following centipede bite. | Cm | 6 | 0.17 | 35 | 0.20 | 0.83 |
| *Scutellaria discolor* Colebr.; MCT-F-081 | Lamiaceae | Tiladiscen | Mida/C | N | H | L | W | *Gastritis* |  | A paste of the leaves is taken at a dose of one spoonful twice daily on an empty stomach for seven days to cure gastritis. | O | 1 | 0.04 | 24 | 0.14 | 0.35 |
| *Senna alata* (L.) Roxb.; MCT-F-062 | Caesalpiniaceae | Dadmardhan | Pui chi/M | N | S | L | W | *Eczema* | 22●,24●,28● | A paste of the leaves is used to treat eczema | O | 1 | 0.03 | 34 | 0.19 | 0.47 |
| *Senna hirsuta* (L.) H.S. Irwin&Barneby; MCT-F-042 | Caesalpiniaceae | Gandhosena | Unknown /C | N | H | L | W | *Dandruff* |  | A paste of the leaves is smeared on the scalp and left for one hour; this is repeated once daily for three days to treat dandruff. | Cm | 1 | 0.03 | 34 | 0.19 | 0.47 |
| *Senna siamea* (Lam.) H.S. Irwin & Barneby; MCT-F-082 | Caesalpiniaceae | Minjiri | Herda pata/M | N | T | L | W | *Gastritis* |  | Leaves are boiled in water and the extract is taken at a dose of one cupful twice daily for fifteen days. | Cm | 1 | 0.04 | 22 | 0.13 | 0.32 |
| *Senna tora* (L.) Roxb.; MCT-F-109 | Caesalpiniaceae | Terasena | Dang geya/M | N | H | L | W | *Jaundice* | 14● | Leaf juice is given at a dose of one cupful daily to treat jaundice. | Cm | 1 | 0.06 | 17 | 0.10 | 0.27 |
| *Sida rhombifolia* L.; MCT-F-130 | Malvaceae | Lalberela | Unknown/M | E | H | WP | W | *Rheumatism* | 23●,25● | An extract of the whole plant is taken to treat rheumatism. | Cm | 1 | 0.04 | 23 | 0.13 | 0.34 |
| *Smilax odoratissima*  Blume; MCT-F-144 | Smilaceae | Gondholata | Khecroba/M | N | S | R | W | *To stop bleeding, dehydration* |  | A paste prepared from the root is used as a haemostatic. Root sap is taken to treat dehydration. | O | 2 | 0.13 | 15 | 0.09 | 0.32 |
| *Solanum erianthum* D.Don; MCT-F-066 | Solanaceae | Eri begun | Taratabba/M | N | S | R | W | *Excessive bleeding after child birth* |  | Sap of the root is taken, using as much as possible until a cure is effected. | Rr | 1 | 0.05 | 19 | 0.11 | 0.29 |
| *Solanum nigrum* L.; MCT-F-146 | Solanaceae | Tit begun | Kheyangeikeci/C | N | H | L | W | *Tonic, liver disorders* | 18●,23●,25●,10♦,17♦ | A decoction of the leaves is taken as a tonic. This same preparation is taken with honey at a dose of one teaspoonful twice daily for five days to treat liver disorders. | A | 2 | 0.17 | 12 | 0.07 | 0.28 |
| *Solanum torvum* Sw.; MCT-F-085 | Solanaceae | Gothbegun | Kuzzu chi/M | N | S | R | W | *Gastritis, toothache* | 19●,28●,24♦ | Juice of the root is taken at a dose of one spoonful twice daily for one month to treat gastritis. The root is chewed for five minutes in cases of toothache. | O | 2 | 0.13 | 15 | 0.09 | 0.32 |
| *Sphaeranthus indicus* L.; MCT-F-102  Least Concern | Asteraceae | Indisag | Crasaing chi/M | N | H | WP | W | *Insect control* |  | The whole plant is kept in the house to control insect parasites on hens. | O | 1 | 0.14 | 7 | 0.04 | 0.15 |
| *Stephania japonica* (Thunb.) Miers; MCT-F-141 | Menispermaceae | Nimuka | Thanda manic/T | N | C | Fr | W | *Stomachache, Liver pain* | 26♦ | A paste of the fruit is taken to cure stomachache and liver pain. | O | 2 | 0.07 | 30 | 0.17 | 0.49 |
| *Sterculia villosa* Roxb.; MCT-F-137 | Sterculiaceae | Udal | Peubi/M | N | T | L | W | *Source of Calcium* | 22♦ | The leaves are cut into small pieces and soaked in water, which is consumed as a source of calcium. | O | 1 | 0.20 | 5 | 0.03 | 0.13 |
| *Streblus asper* Lour.; MCT-F-148 | Moraceae | Shaora | Ounni/M | N | S | L | W | *Toothpowder* | 22♦,28♦ | Powdered leaves are used as a tooth powder. | Cm | 1 | 0.11 | 9 | 0.05 | 0.17 |
| *Terminalia arjuna* (Roxb.*ex* Dc.)Wight & Arn.; MCT-F-073 | Combretaceae | Arjun | Unknown /C | N | T | L, Fr | W | *Fever, dysentery* | 25●,30●,24♦,26♦,28♦ | A decoction of the leaves is taken at a dose of one cupful daily for three days to cure fevers. Powdered fruits, taken with honey, are used to treat dysentery. | Rr | 2 | 0.06 | 34 | 0.19 | 0.54 |
| *Terminalia bellirica* (Gaertn.) Roxb.; MCT-F-069 | Combretaceae | Bohera | Taratabra/M | N | T | R, B | W | *Excessive bleeding during childbirth, fever, cough, cholera* | 25●,27●,28●,22♦,26♦,30♦ | An extract of the root is taken at a dose of one teacupful three times daily to treat excessive bleeding during child birth. Juice of the bark is given to treat fever, cough and cholera. | O | 4 | 0.12 | 34 | 0.19 | 0.68 |
| *Thevetia peruviana* (Pers.) K. Schum.; MCT-F-142 | Apocynaceae | Holdekorobi | Jabafirachi/T | N | T | R | C | *Stomachache, psychological disorder* | 30● | Root sap is taken on an empty stomach at a dose of one teaspoonful twice daily for three days in cases of stomachache. Juice of the root, together with other unknown ingredients, is taken to treat psychological disorders. | O | 2 | 0.07 | 28 | 0.16 | 0.47 |
| [*Tinospora* *sinensis* (Lour.) Merr.](http://www.theplantlist.org/tpl1.1/record/tro-20600675); MCT-F-126 | Menispermaceae | Guloncho | Paralpur/T | N | C | R | W | *Pneumonia* |  | A tablet, prepared from the root and honey, is taken three times daily for one month to treat pneumonia. | Rr | 1 | 0.08 | 12 | 0.07 | 0.21 |
| *Torenia asiatica* L.; MCT-F-071 | Scrophulariaceae | Asiatoren | Unknown /T | E | H | WP | W | *Fever* |  | Juice of the whole plant is taken at a dose of one cupful twice daily for seven days in cases of fever. | Rr | 1 | 0.10 | 10 | 0.06 | 0.19 |
| *Tridax procumbens* (L.) L.; MCT-F-121 | Asteraceae | Tridhara | Unknown /M | N | H | L, WP | W | *Paralysis, cuts and wounds* | 21♦,25♦ | Fresh leaves are baked, then sap of baked leaves is applied to treat paralysis. A paste prepared from whole plants is applied to cuts and wounds. | Cm | 2 | 0.12 | 16 | 0.09 | 0.33 |
| *Tylophora indica* (Burm.f.) Merr.; MCT-F-116 | Asclepiadaceae | Anontomul | Dianerpro/T | N | C | L | W | *Dysmenorrhea* |  | A decoction of leaves is taken to treat dysmenorrhea. | Rr | 1 | 0.07 | 15 | 0.09 | 0.24 |
| *Urena lobata* L.; MCT-F-041 | Malvaceae | Batapuran | Poppy/M | N | H | R | W | *Cut and wounds, tonsillitis* | 20●,18♦,19♦,25♦ | Sap of the root is applied to the affected areas in cuts sustained from iron objects. The juice of the root is given to treat tonsillitis. | Cm | 2 | 0.09 | 21 | 0.12 | 0.39 |
| *Vitex negundo* L.; MCT-F-035 | Verbenaceae | Nishinda | Soyin ma pata/T | N | S | L | W | *Coughs & colds, vitiligo* | 12●,20●,30●,21♦,23♦ | A paste of the leaves is smeared onto affected areas to treat vitiligo. Juice of the leaves is taken at a dose of one cupful daily to treat vitiligo. This same preparation is taken along with honey to treat coughs and colds. | A | 2 | 0.09 | 23 | 0.13 | 0.41 |
| *Woodfordia fruticosa* (L.) Kurz; MCT-F-058  Lower risk/Least Concern | Lythraceae | Rangkat | Taiyangshi/M | N | H | L, Fw | W | *Dysentery; Excessive bleeding during menstruation.* | 25● | Leaf sap, along with garlic (*Allium sativum*) is taken at a dose of one spoonful three times daily for five days to treat dysentery. An extract of the flowers is taken (as much as possible) to stop excessive bleeding during menstruation. | Rr | 2 | 0.09 | 21 | 0.12 | 0.39 |
| *Zingiber officinale* Roscoe; MCT-F-077 | Zingiberaceae | Ada | Ke/C; Kyang/M; aara/T | N | H | Rz | C | *Flatulence , gastritis, carminative, abdominal pain, coughs and colds.* | 11●,17●,26●,28●,30●,16♦ | The rhizome, smashed along with a small quantity of table salt and honey, is taken to treat coughs and colds (/M). Juice of the rhizome, with black cumin (*Nigella sativa*) is taken twice daily for seven days to treat flatulence, abdominal pain and gastritis (/C). A paste of the rhizome, along with rice, is given as a carminative (/T). | Cm | 5 | 0.42 | 12 | 0.07 | 0.50 |
| *Zingiber zerumbet* (L.) Roscoe *ex* Sm.; MCT-F-083 | Zingiberaceae | Bonada | Podogro/M | N | H | Rz | W | *Gastritis* |  | A paste of the rhizome, along with salt, is taken to cure gastritis | O | 1 | 0.05 | 21 | 0.12 | 0.32 |
| *Ziziphus mauritiana* Lam.; MCT-F-115 | Rhamnaceae | Kul | Joci/C; Jim Bang/M | N | T | R, L | C | *Menstrual disorders, scabies, ulcers* | 20●,25●,30●,8♦,23♦,28♦ | An infusion of leaves is prepared with honey, and taken orally every morning for one month to treat ulceration. This same preparation is taken prior to menstruation, in cases of ongoing menstruation difficulties (/C). A paste of the leaves is rubbed onto lesions to treat scabies (/M). | Cm | 3 | 0.14 | 21 | 0.12 | 0.46 |

Abbreviations: FoD = Frequency of Distribution; UR = Use Reports; UV = Use Value; FC = Frequency of Citation; RFC = Relative Frequency of Citation; RI = Relative Importance Index

/M= Marma Community; /C= Chak Community; /T= Tanchayanga Community; L= Leaves; S= Stem; R= Root; Fw = Flower; Fr = Fruit; Bb = Bulb; Rz = Rhizome; P = petiole; WP = Whole Plant; Tb = Tuber; Sd = Seed; B = Bark; T = Tree; H = Herb; Sb = Shrub; C = Climber; N = Native; E = Exotic; A = Abundant; Cm= Common; O = Occasional; Rr = Rare.

●= similar use; ♦= dissimilar use;1= ; 2= ; 3= ; 4= ; 5= ; 6= ; 7= ; 8= ; 9= ; 10= ; 11= ; 12= ; 13= ; 14= ; 15= ; 16= ; 17= ; 18= ; 19= ; 20= ; 21= ; 22= ; 23= ; 24= ; 25= ; 26= ; 27= ; 28= Uddin et al., 2013; 29=; 30=
